# Protocol for single-nucleus ATAC sequencing and bioinformatic analysis in frozen human brain tissue

**DOI:** 10.1016/j.xpro.2022.101491

**Published:** 2022-06-17

**Authors:** Zechuan Shi, Sudeshna Das, Samuel Morabito, Emily Miyoshi, Vivek Swarup

**Affiliations:** 1 Department of Neurobiology and Behavior, University of California, Irvine, Irvine, CA, USA; 2 Mathematical, Computational and Systems Biology Program, UC Irvine, Irvine, CA, USA; 3 Institute for Memory Impairments and Neurological Disorders (MIND), Irvine, CA, USA

**Keywords:** Bioinformatics, Sequence analysis, Health Sciences, Genomics, Sequencing, Neuroscience

## Abstract

Single-nucleus ATAC sequencing (snATAC-seq) employs a hyperactive Tn5 transposase to gain precise information about the cis-regulatory elements in specific cell types. However, the standard protocol of snATAC-seq is not optimized for all tissues, including the brain. Here, we present a modified protocol for single-nuclei isolation from postmortem frozen human brain tissue, followed by snATAC-seq library preparation and sequencing. We also describe an integrated bioinformatics analysis pipeline using an R package (ArchRtoSignac) to robustly analyze snATAC-seq data.

For complete details on the use and execution of this protocol, please refer to Morabito et al. (2021).

## Highlights

• Frozen human brain dissection and single nucleus isolation for snATAC-seq
• Library preparation and quality control for snATAC-seq from nuclei of frozen human brain
• snATAC-seq analysis protocol and pipeline conversion with ArchRtoSignac package
• Clustering with reference mapping and differential accessible peaks identification

## Abstract (editor highlights)

Publisher’s note: Undertaking any experimental protocol requires adherence to local institutional guidelines for laboratory safety and ethics.

## Graphical abstract

**Figure:**
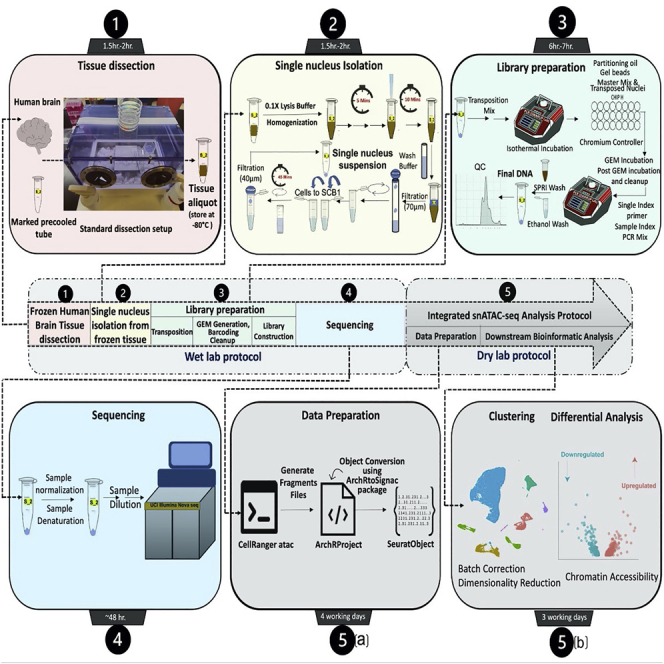

## Before you begin

Assay for transposase-accessible chromatin coupled with high-throughput sequencing (ATAC-seq) is a simple, fast, and sensitive method for mapping chromatin accessibility genome-wide and is therefore considered as an alternative to other methods like DNase-seq or MNase-seq (Buenrostro et al., 2015). ATAC-seq works well across many cell types and species and requires only 50,000 cells or less to capture regulatory elements (Shashikant and Ettensohn, 2019). However, bulk measurements of chromatin accessibility limit the precise understanding of how specific cell-types contribute to overall disease etiology (Fang et al., 2021). Recent innovations in single-cell genomics have enabled scalable profiling of chromatin with cellular or subcellular resolution using single nucleus ATAC-seq (snATAC-seq) (Rai et al., 2020; Ziffra et al., 2021). The typical bulk ATAC or snATAC-seq protocol requires nuclei extracted from fresh cells and tissues, due to the significant disruptive effect of freezing on chromatin integrity and structure. However, intensive effort has been made to introduce modifications in the snATAC-seq protocol to allow nuclei extraction from frozen tissues, including frozen mammalian brains. The current protocol provides a simple yet highly reproducible pipeline for isolation of single nuclei from frozen postmortem human brains followed by library preparation for snATAC-seq and downstream bioinformatics analysis. The tissue dissection protocol and modified single nucleus isolation protocol together give rise to enough intact nuclei from the frozen brain to achieve targeted nuclei recovery for further processing. Additionally, our integrated bioinformatic protocol using both ArchR and Signac pipelines (Granja et al., 2021; Stuart et al., 2021) provides an approach, specifically an R package (ArchRtoSignac), to convert an ArchRProject to a Signac SeuratObject. This conversion R package keeps the 501 bp fixed-width open chromatin regions in the peak matrix generated by ArchR for chromatin accessibility analysis and provides the option to access additional advanced secondary analyses through Signac, in addition to the functions in the ArchR pipeline. Due to the inherent sparsity of snATAC-seq data and the heterogeneity of human samples, a minimum of 2000–3000 nuclei per sample with four biological replicate samples per group is highly recommended for the analysis of open chromatin changes. In summary, the modified wet lab protocol is robust, simple, and capable of generating high-quality chromatin accessibility data from frozen human tissue. The integrated dry lab protocol provides a data format conversion to take advantage of two popular snATAC-seq analysis pipelines, ArchR and Signac, which is more robust than using the two pipelines separately.

### Institutional permissions

Human postmortem brain samples (prefrontal cortex, PFC) were obtained from UCI MIND’s Alzheimer’s Disease Research Center (ADRC) tissue repository under the Institutional Review Board (IRB) of UCI. All participants, or participants’ legal representative, provided written informed consent for the study.

### Preparation

#### Part one: Wet lab preparation

**Timing: ∼1 h (for preparationof frozen human brain tissue dissection)**

**Timing: ∼1 h (for preparationof nuclei isolation)**

**Timing: ∼2 h(forlibrary preparation)**

1. Frozen human brain tissue dissection.
  Please check materials and equipment section (Materials needed for frozen human brain dissection) for a complete list of required reagents and consumables.
  a. Clean the dissection box with 10% bleach (contact time 10 min) followed by freshly prepared 70% ethanol. Place the dissection box on absorbent pads.
  b. Prepare the −80°C box where the 1.5 mL tubes (nuclease-free and LoBind tubes e.g., 1.5 mL Eppendorf tubes, Catalog No. 022431021) will be kept by labeling it and putting it on dry ice outside the dissection box.
  c. Mark and prechill all the 1.5 mL tubes.
  d. Prechill nuclease-free spatula, petri-dishes, and tissue forceps (preferably with teeth on the tips of the forceps for more secure hold) in dry ice.
  e. Weigh a few 1.5 mL Eppendorf tubes to get an idea of the weight.
2. Nuclei isolation from frozen human brain tissue for snATAC-seq.
  a. Set the centrifuge temperature at 4°C.
  b. Mark all the 1.5 mL and 15 mL tubes.
  c. Thaw/equilibrate the reagents to room temperature (20°C–25°C) or specific thawing temperature (e.g., incubate digitonin at 65°C to dissolve precipitates).
  d. Prepare fresh Wash Buffer (1×), 1× Lysis Buffer, Lysis Dilution Buffer, 0.1× Lysis Buffer, Sucrose Cushion Buffer I and Diluted Nuclei Buffer (Please see the ‘buffers for single nucleus isolation’ section for required reagents and buffer recipes). Keep the buffers on ice before use. Do not store the buffers overnight (>6 h).
  e. Prewet 70 μm Cell Strainer with sterile and nuclease-free 1× PBS.
3. Library preparation:
  Please refer to the detailed kits instruction for exact guidelines and preparation. However, few common preparations are listed below.
  a. Thaw/equilibrate the buffers, gel beads, primers to room temperature (20°C–25°C) or specific thawing temperature (Please refer to Chromium Next GEM Single Cell ATAC Reagent Kits v1/v1.1 user manual).
    ***Note:*** A few reagents, including cleanup buffer, require special thawing conditions like a 10 min incubation at 65°C with max speed (∼1400 rpm) on a ThermoMixer.
  b. Place enzymes on ice 5–10 min before adding to the reaction mixture.
  c. Set temperatures and cycle numbers in the PCR machines (always double-check before incubating the reaction mixture).
  d. Mark all the tubes and place those on ice if needed.
  e. Properly mix the beads (SPRI and Dynabeads) before use.
  f. Prepare glycerol solution (for <8 samples).
  g. Thaw, vortex, and centrifuge gel beads before loading into the chip.
  h. Prepare 80% ethanol (fresh) before use.
  i. Prepare reagents/working solutions (as required) for quantifying DNA (by *Qubit Fluorometer*) and measuring average fragment length (by *TapeStation*).

#### Part two: Dry lab preparation

Installing cellranger-atac on a local PC or high-performance computing cluster.

We can install cellranger-atac software on a local desktop/Linux environment or on a high-performance computing cluster. Full instructions for downloading and installing the software and reference data package can be found on the 10× Genomics webpage.

4. A proper reference is needed for read alignment. For example, if a human sample reference is needed for snATAC-seq, please download and use the human genome reference (GRCh38) dataset from cellranger: refdata-cellranger-arc-GRCh38-2020-A-2.0.0.tar.gz.
5. Please unzip it for the following use:

>tar -xvf refdata-cellranger-arc-GRCh38-2020-A-2.0.0.tar.gz

Installing ArchRtoSignac package for data format conversion.

We made an R package with several wrapper functions to make the object conversion from ArchRProject to SeuratObject simplified.

6. Install the ArchRProject to Signac SeuratObject conversion function by running the following code:

>if(!require(devtools)){

 install.packages("devtools") # If not already installed

}

>devtools::install_github('swaruplabUCI/ArchRtoSignac')

>library(ArchRtoSignac)

# Please load your choice of reference.

>library(EnsDb.Hsapiens.v86) # Choice of reference library

***Note:*** To analyze snATAC-seq data, ArchR and Signac packages are required, and they are required for the use of ArchRtoSignac package. However, due to the complexity of the programming environment, we suggest constructing one isolated conda environment with ArchR, Signac and other dependencies to avoid package conflicts for snATAC-seq analysis. After installing ArchRtoSignac package, we provide a quick way to check if all required packages are installed and load those available packages automatically. Please refer to the ArchRtoSignac GitHub page for detailed instructions for installation and construction of an isolated conda environment.

# Packages we need for ArchRtoSignac

> packages <- c("ArchR","Seurat", "Signac","stringr")

> loadinglibrary(packages)

# For example: If the required R package is not available, a “Package not found” message will remind the researcher to install the package. Otherwise, the R package will be loaded.

# Loading required package: Signac

# [1] " Package Signac not found. Please Install the Package!!"

# On contrary, if the required R package is available, the following message will display:

# [1] "Loading Package: stringr"

# [1] "Package: stringr -- Loaded Successfully"

## Key resources table

**Table:** 

REAGENT or RESOURCE	SOURCE	IDENTIFIER
**Chemicals, peptides, and recombinant proteins**		

Tris-HCl (1 M)^∗^	Sigma	T2194-100ML
NaCl^#^	Sigma	S9888-500g
MgCl2 (1 M) ^#^	Thermo Fisher Scientific	AM9530G
BSA (10%)^#^	Sigma	A1595-50ML
Tween-20 (10%)^#^	Bio-Rad	161-0781
NP40 Substitute (10%)^#^	Sigma	98379
Nuc Free Water^#^	Thermo Fisher Scientific	AM9932
Digitonin (5%)^∗^	Thermo Fisher Scientific	BN2006
Nuclei Pure Prep Nuclei Isolation Kit^∗^	Sigma	NUC-201
Chromium Next GEM Single Cell ATAC Library Kit v1.1^∗^	10× Genomics	PN-1000163 (store at −20°C)
Chromium Next GEM Single Cell ATAC Gel Bead Kit v1.1^∗^	10× Genomics	PN-1000159 (store at −80°C)
Single Index Kit N Set A^∗^	10× Genomics	PN-1000212
DynabeadsTM MyOneTM SILANE^∗^	10× Genomics	PN-2000048 (store at 4°C)
Recovery Agent^∗^	10× Genomics	220016
Partitioning Oil^∗^	10× Genomics	2000190
Gasket^∗^	10× Genomics	370017
Chromium Next GEM Chip H^∗^	10× Genomics	2000180
Bleach (Pure Bright Liquid Germicidal Ultra) ^#^	Thomas Scientific	KIK 949975
Ethanol, Absolute (200 Proof) ^#^	Sigma	E7023-500ML
SPRIselect Reagent Kit (contains SPRI beads) ^∗^	Beckman Coulter	B23318
Glycerin (glycerol), 50% (v/v) Aqueous Solution^∗^	RICCA Chemical Company	3290-32
Qiagen Buffer EB^∗^	QIAGEN	19086
Low TE Buffer^∗^	Thermo Fisher Scientific	12090-015

**Other**		

MasterCycler Pro Thermal Cycler^#^	Eppendorf	North America 950030010 International 6321 000.019
Vortex Mixer^#^	VWR	10153-838
Fisherbrand™ motorized tissue grinder^∗^	Thermo Fisher Scientific	12-1413-61
Fisherbrand™ RNase-Free Disposable Pellet Pestles^∗^	Thermo Fisher Scientific	12-141-364
Divided Polystyrene Reservoirs^#^	VWR	41428-958
Tips^∗^	Rainin	30389240 (200 μL); 30389213 (1 mL); 30389226 (20 μL)
TempAssure PCR 8-tube strip^∗^	USA Scientific	1402-4700
DNA LoBind Tubes, 1.5 mL^#^	Eppendorf	022431021
DNA LoBind Tubes, 2.0 mL^#^	Eppendorf	022431048
Mini Centrifuge^#^	Thermo Fisher Scientific	C1012
Eppendorf ThermoMixer C^#^	Eppendorf	5382000023/2231000574
Drierite with indicator, 8 mesh^#^	Thermo Fisher Scientific (Acros Organics)	21909-0020
Pre-Separation Filters (70 μm) ^#^	Miltenyi Biotec	130-095-823
Flowmi® Cell Strainers (40 μm) ^#^	Sigma	H13680-0040
4200 TapeStation^∗^	Agilent	G2991AA
High Sensitivity D1000 ScreenTape^∗^	Agilent	5067-5584
High Sensitivity D1000 Reagents^∗^	Agilent	5067-5585
10× Vortex Adapter^∗^	10× Genomics	120251(orderable), 330002 (Item)
Chromium Next GEM Secondary Holder^∗^	10× Genomics	1000195(orderable), 3000332 (Item)
10× Magnetic Separator^∗^	10× Genomics	120250 (orderable), 230003 (Item)
NovaSeq6000^#^	Illumina	N/A
Qubit™ 4 Fluorometer^∗^	Thermo Fisher Scientific	Q33238
Qubit™ 1× dsDNA HS Assay Kit^∗^	Thermo Fisher Scientific	Q33231
Countess™ 3 FL Automated Cell Counter^#^	Thermo Fisher Scientific	AMQAF2000
DAPI^#^	Thermo Fisher Scientific	R37606
Countess™ Cell Counting Chamber Slides^#^	Thermo Fisher Scientific	C10283/C10228

**Biological samples**		

Adult postmortem human brain Age range: 74–90+ Sex: Male and Female	UCI MIND’s Alzheimer’s Disease Research Center (ADRC) tissue repository	N/A

**Deposited data**		

snATAC-seq data (postmortem frozen human brain tissue)	Synapse	Synapse ID: syn22079621
snRNA-seq data (postmortem frozen human brain tissue)	Synapse	Synapse ID: syn22079621

**Software and algorithms**		

cellranger-atac	10× Genomics	https://support.10×genomics.com/single-cell-atac/software/pipelines/latest/what-is-cell-ranger-atac
R Version 4.1.1	R Project	https://www.r-project.org
ArchR	(Granja et al., 2021)	https://www.archrproject.com/index.html
Signac	(Stuart et al., 2021)	https://satijalab.org/signac/index.html
Seurat	(Ntranos et al., 2019)	https://satijalab.org/seurat/
Monocle3	(Cao et al., 2019)	https://cole-trapnell-lab.github.io/monocle3/
Cicero	(Pliner et al., 2018)	https://cole-trapnell-lab.github.io/cicero-release/
GREAT	(McLean et al., 2010)	http://great.stanford.edu/public/html/
ArchRtoSignac	Current Manuscript	https://github.com/swaruplabUCI/ArchRtoSignac/ DOI: 10.5281/zenodo.6612020

∗Critical Reagents/Instruments/Plasticwares/others.

#These are the **Reagents/Instruments/Plasticwares** that we used, although we can only speculate, we do not expect the use of alternative (compatible) reagents/instruments/ plasticwares to influence the efficiency of the assay. Please refer to Chromium Next GEM Single Cell ATAC Reagent Kits v1/v1.1 user manual for more information about alternatives.

## Materials and equipment

### Wet lab section

**Materials needed for frozen human brain dissection:** 10% bleach (make fresh every week), 70% ethanol (make fresh every week), scalpel (disposable), Kimwipes, absorbent pad, forceps, spatula, 10 cm petri-dishes, dry ice, Drierite indicating absorbent, storage box for 1.5 mL tubes in −80°C, insulated gloves, nitrile gloves, scale (sensitive), nuclease-free and DNA LoBind 1.5 mL tubes (pre-labeled).

### Buffers for single nucleus isolation

**CRITICAL:** Please prepare fresh buffers before use and keep the buffers on wet ice before adding to the tissue/nuclei. Do not store the buffers more than 6 h (360 min) on ice.

***Note:*** We need 1.5 mL Wash Buffer /sample (Please make 2 mL/sample).

***Note:*** Required amount of 0.1× Lysis Buffer is 0.5 mL/Sample.

***Note:*** Required amount of Sucrose Cushion Buffer I is 2.8 mL/sample (Please make 3 mL/sample).

***Note:*** Required amount of Diluted Nuclei Buffer depends on the number of isolated nuclei (Please make 1 mL/sample).

**Table:** Wash buffer

Component (storage)	Stock	Final	8 mL (for 4 samples)
Tris-HCl pH 7.4 (RT)	1 M	10 mM	80 μL
NaCl (RT)	5 M	10 mM	16 μL
MgCl_2_ (RT)	1 M	3 mM	24 μL
BSA (4°C)	10%	1%	800 μL
Tween-20 (RT)	10%	0.10%	80 μL
Nuc Free water (RT)	–		7 mL

Store on wet ice or at 4°C (not more than 6 h) before use.

**Table:** 1× lysis buffer

Component (storage)	Stock	Final	2 mL (only 200 μL is required for 4 samples)
Tris-HCl pH 7.4 (RT)	1 M	10 mM	20 μL
NaCl (RT)	5 M	10 mM	4 μL
MgCl_2_ (RT)	1 M	3 mM	6 μL
Tween-20 (RT)	10%	0.10%	20 μL
Nonidet P40 Substitute (RT)	10%	0.10%	20 μL
Digitonin (Storage is 4°C; Incubate at 65**°**C to dissolve precipitate before use)	5%	0.01%	4 μL
BSA (4°C)	10%	1%	200 μL
Nuc Free water (RT)	–		1.732 mL

Store on wet ice or at 4°C (not more than 6 h) before use.

**Table:** Lysis dilution buffer

Component (storage)	Stock	Final	2 mL (for 4 samples)
Tris-HCl pH 7.4 (RT)	1 M	10 mM	20 μL
NaCl (RT)	5 M	10 mM	4 μL
MgCl_2_ (RT)	1 M	3 mM	6 μL
BSA (4°C)	10%	1%	200 μL
Nuc Free water (RT)	–		1.732 mL

Store on wet ice or at 4°C (not more than 6 h) before use.

**Table:** 0.1× lysis buffer

Component (storage)	Stock	Final	2 mL (for 4 samples)
1× Lysis Buffer (ice)	1×	0.1×	200 μL
Lysis Dilution Buffer (ice)			1.8 mL

Store on wet ice or at 4°C (not more than 6 h) before use.

**Table:** Sucrose cushion buffer I

Component (storage)	Stock	Final	12 mL (for 4 samples)
Nuclei PURE 2 M Sucrose Cushion Soln. (4°C; included in Nuclei Pure Prep Nuclei Isolation Kit)	–	–	10.8 mL
Nuclei PURE Sucrose Cushion Buffer (4°C; included in Nuclei Pure Prep Nuclei Isolation Kit)	–	–	1.2 mL

Store on wet ice or at 4°C (not more than 6 h) before use.

**Table:** Diluted nuclei buffer

Component (storage)	Stock	Final	2 mL (for ∼ 4 samples)
Nuclei Buffer 20× (−20°C; included in Single Cell ATAC Library Kit)	20×	1×	100 μL
Nuc Free water (RT)			1900 μL

Store on wet ice or at 4°C (not more than 1 h) before use.

### Data analysis section

• **snATAC-seq Fastq data processing.** The required package can be installed on your local computer or HPC. While a different version of cellranger-atac would work, we use cellranger-atac v2.0.0.
  • cellranger-atac (v2.0.0.).

• **R software and required packages.** ArchR and Signac are software for analyzing, interpreting, and exploring single-nucleus chromatin datasets. For this protocol, we used R version 4.1.1, but we acknowledge that other versions of R and the associated packages are compatible with ArchR and Signac. We suggest creating a conda environment specifically for this protocol to install the required software, thereby neatly containing all software for this protocol in one directory and ensuring that the software versions do not conflict with software required by other research projects. We used the following packages at the indicated versions when writing this protocol:
  • ArchR (v1.0.1).
  • Signac (v1.5.0).
  • Seurat (v4.1.0).
  • SeuratWrapper (v0.3.0).
  • Monocle3 (v1.0.0).
  • Cicero (v1.3.4.11).
  • chromVAR (v1.16.0).
  • rGREAT (v1.26.0).
  • ArchRtoSignac (v0.0.0.9000).
  • Harmony (v0.1.0).
  • EnsDb.Hsapiens.v86 (v2.99.0).
  • dplyr (v1.0.8).
  • ggplot2 (v3.3.5).
    Automatically attached R packages when loading the above packages:
  • Ensembldb (v2.18.3).
  • AnnotationFilter (v1.18.0).
  • GenomicFeatures (v1.46.4).
  • AnnotationDbi (v1.56.2).
  • SeuratObject (v4.0.4).
  • magrittr (v2.0.2).
  • rhdf5 (v2.38.0).
  • Matrix (v1.4-0).
  • data.table (v1.14.2).
  • SummarizedExperiment (v1.24.0).
  • Biobase (v2.54.0).
  • GenomicRanges (v1.46.1).
  • GenomeInfoDb (v1.30.1).
  • IRanges (v2.28.0).
  • S4Vectors (v0.32.3).
  • BiocGenerics (v0.40.0).
  • MatrixGenerics (v1.6.0).
  • matrixStats (v0.61.0).

• An additional package required by ArchR, Model-based Analysis for ChIP-Seq (MACS), was installed through conda environment. MACS is used for peak calling in snATAC-seq analysis. Installation instructions for conda can be found in conda’s latest user guide.
  • MACS (v2.2.7.1).

> conda install -c bioconda macs2

• Hardware.
  • Local – Memory: 8 or 16 GB required; Processors: 1 required for small datasets or a pilot study.
  • Computational Cluster – Memory: 32 or 64 GB suggested; Processors: > 8 recommended for parallel processing and large datasets.

## Step-by-step method details

### Part one: Wet lab protocol

**Timing: 7–10 min for each sample dissection. Additionally, an interval of 3–4 min between each sample is required to prechill the accessories**

**Timing: 1.5–2 hfor nuclei isolation**

**Timing: ∼5 min for calculation and 5 min for dilution**

**Timing: ∼6 h for 4 major steps including Transposition, GEM (Gel Beads- in-emulsion) Generation & Barcoding, Post GEM Incubation Cleanup and Library Construction**

**Timing: 70–80 minfor transposition**

**Timing: 1.5 hfor GEM generation & barcoding**

**Timing: ∼50 minfor post GEM incubation cleanup**

**Timing: ∼2 hfor library construction**

**Timing: 2 working daysfor sequencing**

The objective of part one is to isolate nuclei from frozen human brain samples and process it for single-nucleus ATAC-seq library preparation and sequencing.

**Figure 1:**
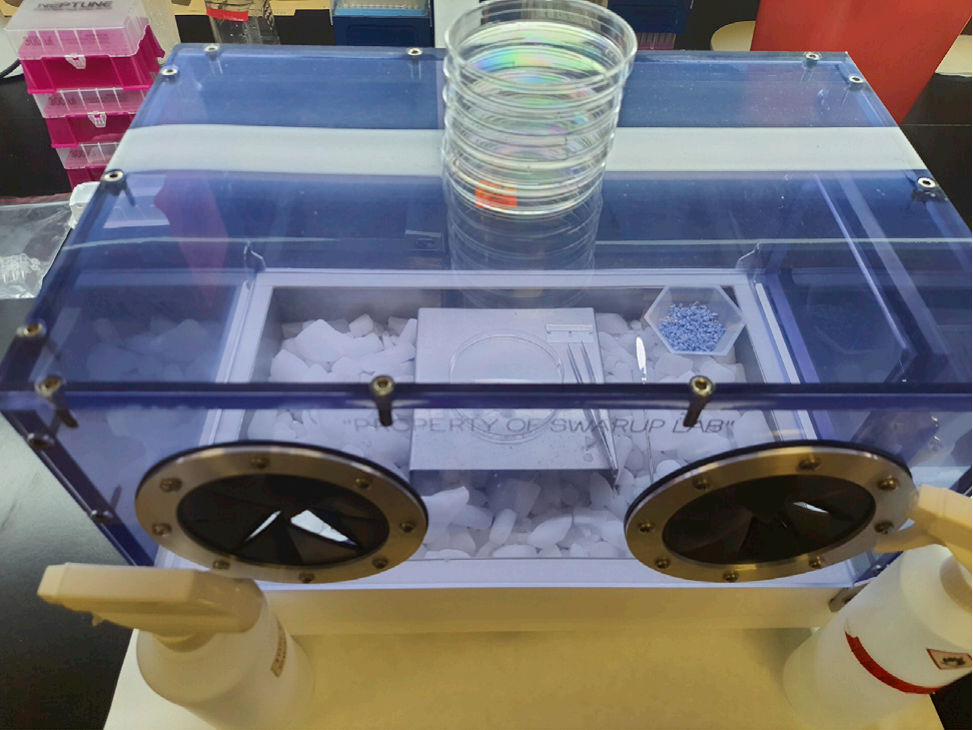
Dissection box setup The precleaned box is placed on an absorbent pad and the inner surface of the insulated box is fully covered with dry ice. All required things including a spatula, petri-dishes, labeled 1.5 mL tubes and forceps are precooled in dry ice. 50–100 mg of Drierite absorbent is also placed within the box to reduce the moisture level.

**Figure 2:**
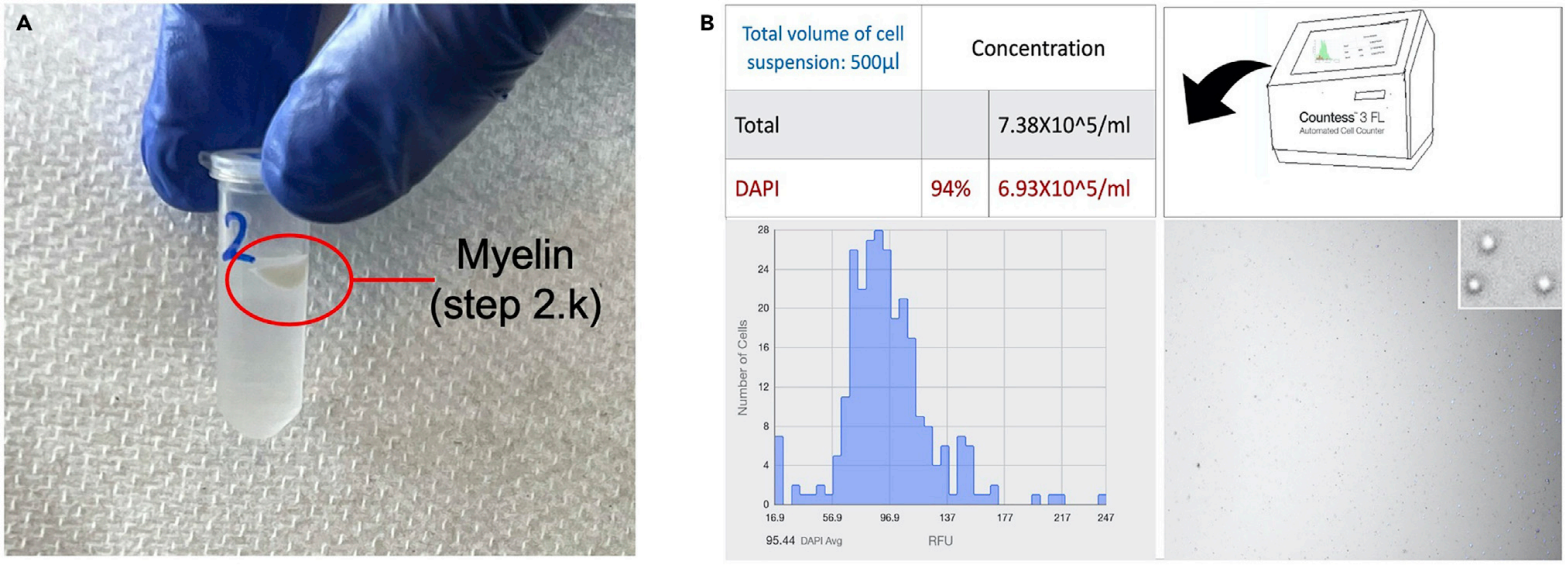
Representative images of myelin and isolated nuclei (A) Myelin content (after step 2.k; a white sticky layer as shown here) at the top and sidewall of the tube after centrifugation at 13000 g for 45 min at 4°C. (B) Isolated single nucleus counting (step 2.q) by *Countess 3 FL Automated Cell Counter*. After filtration with a 40 μm Flowmi Cell Strainer and combining the aliquots (steps 2.o and 2.p), 1 μL of DAPI was added to 10 μL of cell suspension and loaded to the slides. The visual field indicates optimal cell lysis (≥ 85% DAPI count) with high quality, round nuclei and absence of cell debris or large clumps.

***Note:*** Verify the compatibility of the sequencer with the kit and proceed accordingly.

***Note:*** A representative information sheet for sequencing a sample (using the above protocol and library produced by 10× Genomics kit) is shown below for a better understanding.

1. Frozen human brain tissue dissection:
  ***Note:* Required amount of tissue** is 50(±5) mg flash frozen brain tissue.
  ***Note:*** This step is required to aliquot the exact amount of tissue for the assay.
  **CRITICAL:** Do not thaw the tissue at any point. Use enough dry ice to fully cover the tubes containing tissue.
  a. Fill the dissection box with dry ice, making sure that the surface is completely covered and full of dry ice as shown in Figure 1.
    ***Note:*** Wait for at least 15–20 min after the initial filling for the chamber to cool down completely.
  b. Keep 50–100 mg of Drierite absorbent (with indicator; 8 mesh) within the box to absorb excess moisture.
  c. Dissect each sample carefully in the dissection box on a petri-dish. The top and bottom of each dish can be used for separate samples.
  d. Aliquot tissue in the prechilled 1.5 mL tube and then quickly weigh on the scale.
    ***Note:*** Required amount of tissue per sample is 50(+/- 5) mg (around 0.5 cm size) flash frozen brain tissue.
  e. Immediately place the samples on dry ice and finally in the storage box (already on dry ice).
  f. Wipe the spatula and forceps with 70% ethanol. Discard the petri-dish in biohazard bins.
  g. At the end of the day, leave the dry ice in the box, and the next morning, clean the box with 10% bleach (contact time 10 min) and then 70% ethanol.
    **Pause point:** Store the tissue samples at −80°C or proceed to the next step (step 2.a).

2. Nuclei isolation from frozen human brain tissue for snATAC-seq.
  ***Note:*** This step is required for preparing a single nucleus suspension (of optimum concentration and quality) from the desired brain tissue.
  ***Note:* Reagents needed** include freshly prepared Wash Buffer (1×), freshly prepared 1× Lysis Buffer, Lysis Dilution Buffer, freshly prepared 0.1× Lysis Buffer, Sucrose Cushion Buffer I, Diluted Nuclei Buffer (See the ‘buffers for single nucleus isolation’ section for required reagents and buffer recipes).
  **CRITICAL:** Set the centrifuge temperature at 4°C before starting the experiment (before step 2.a).
  **CRITICAL:** Do not thaw the tissue before adding the Lysis Buffer.
  **CRITICAL:** Make fresh Wash Buffer and Lysis Buffer. Do not store.
  **CRITICAL:** Do not start with more than 8 samples.
  **CRITICAL:** Do not freeze isolated nuclei following nuclei isolation.
  a. Add 500 μL chilled 0.1× Lysis Buffer to the frozen brain tissue and immediately homogenize 15 times using a pellet pestle (Fisherbrand™ Pellet Pestle™ Cordless Motor with RNase-Free Disposable Pellet Pestles, Catalog No.12-141-364).
    **CRITICAL:** Avoid bubble formation during this step.
  b. Incubate the lysate for 5 min on ice.
  c. Pipette mix 10 times. You can use a wide-bore pipette tip if needed.
  d. Incubate the lysate for 10 min on ice.
  e. Add 500 μL Wash Buffer (chilled) to each tube and pipette mix 5–7 times.
  f. Pass the suspension through a 70 μm Cell Strainer (prewet the strainer with 1× nuclease-free and sterile PBS) into a 15 mL marked and precooled tube.
  g. Add 1.8 mL Sucrose Cushion Buffer I to the 15 mL tube and pipette mix 10 times.
  h. Prepare two sucrose gradients by adding 500 μL Sucrose Cushion Buffer I to two new marked 2 mL tubes.
  i. The nuclei suspension (2.8 mL) of each sample will be divided into 2 tubes (2 mL tubes with Sucrose Cushion Buffer I) in this step.
  j. Carefully layer 1.4 mL of nuclei suspension (half of the total volume, i.e., 2.8 mL) to the top of each 2 mL tube containing Sucrose Cushion Buffer I. Do not mix.
  k. Centrifuge the tubes (containing nuclei suspension and Sucrose Cushion Buffer I) at 13000 g for 45 min at 4°C.
  l. Check the myelin content (a white sticky layer as shown in Figure 2A) at the top and sidewall of the tube and carefully remove the myelin (if needed), using 1 mL pipette tips or a sterile and nuclease free spatula, without disturbing the pellet.
  m. Carefully remove the supernatant leaving 100 μL in each tube and resuspend the nuclei pellet by gentle pipetting (∼10 times). Do not vortex the cell suspension for mixing.
  n. Add 500 μL Wash Buffer and gently pipette mix 8–10 times or until nuclei are completely resuspended.
  o. Carefully pass the resuspended nuclei through a 40 μm Flowmi Cell Strainer into a new 1.5 mL tube.
  p. Combine the 2 aliquots of the same sample at this point. Total volume (in the new 1.5 mL tube) after combining both the filtered cell suspension (of same sample) should be ∼1200 μL.
  q. Determine the nuclei concentration using a cell counter. Take 10 μL of the nuclei suspension and 1 μL of DAPI or trypan blue (please check the amount to be added) for the counting.
  r. Centrifuge the tube with cell suspension (from step n) at 500 g for 5 min at 4°C.
  s. Remove the supernatant without disturbing the pellet.
  t. Resuspend the pellet (final nuclei pellet) in chilled Diluted Nuclei Buffer.
    ***Note:*** Resuspend the pellet in nuclei buffer using the concentration table provided in the kit (Chromium Next GEM Single Cell ATAC Reagent Kits v1/v1.1) manual.

3. Dilution of Nuclei Stock:

***Note:* Total no. of nuclei loaded onto the Chromium chip** is 10,000–15,000.

***Note:* Total volume needed** = 5 μL (for 10× Genomics kit). Please double check the specific kit user manual to ensure correct volume for your kit.

***Note:* Recovery efficiency factor** = 1.53 (The recovery efficiency factor is determined empirically by 10× Genomics; for scATAC reagent kit, the value is 1.53).

***Note:* Targeted Nuclei Recovery =** Total number of nuclei desired for the experiment, and is different from the actual number of nuclei loaded.

$$Volume\;of\;Nuclei\;Stock\;(\mu \text{L})=\frac{\left(\text{Targeted }\;\text{Nuclei}\;\text{Recovery }\times 1.53\right)}{\text{Nuclei }\;\text{Stock }\;\text{Concentration }\;(\text{nuclei}/\text{μL})}$$

VolumeofDilutedNucleiBuffer(μL)=5μl − volume of Nuclei Stock (μL)

4. Library preparation:
  **For library preparation, please refer to the kit’s detailed instructions.** We use the Chromium Next GEM Single Cell ATAC Reagent Kits v1/v1.1.
  ***Note:*** For instrument and accessories needed, please refer to the kit user guide.
  ***Note:*** For reagents, plasticware and kits needed, please refer to the kit user guide.
  a. Transposition:
    ***Note:*** This step is required for fragmentation of the DNA in open regions of chromatin and addition of adapter sequences.
    i. Prepare and add Transposition Mix (see the detailed instruction in the kit user manual) to a tube.
    ii. Add the calculated volume of Nuclei Stock and Diluted Nuclei Buffer to the tube containing Transposition Mix and gently pipette mix.
      ***Note:*** Check the kit’s instruction for the total volume.
    iii. Incubate the tubes in a thermal cycler (please refer to the user guide for the incubation temperatures and time required).
  b. GEM Generation & Barcoding.
    ***Note:*** Objective of this step includes generation of GEMs (by combining barcoded Gel Beads, transposed nuclei, Master Mix, and Partitioning Oil on a Chromium Next GEM Chip H), thermal cycling of the GEMs (for production of 10× barcoded single-stranded DNA) and breaking the GEMs for recovering the pooled fractions.
    Protocol:
    i. Prepare Master Mix with the kit reagents.
    ii. Assemble Chromium Next GEM Chip H.
    iii. Load Chromium Next GEM Chip H with Master Mix and Transposed Nuclei in row labeled 1.
    iv. Load Chromium Next GEM Chip H with Gel Beads in row labeled 2.
    v. Load Chromium Next GEM Chip H with partitioning oil in row labeled 3.
    vi. Run on the Chromium Controller.
    vii. Transfer GEMs to a tube strip.
    viii. Incubate GEMs in a thermal cycler (please refer to the user guide for the required incubation temperatures and time).
      **Pause point:** Store at 15°C for up to 18 h or at −20°C for up to 1 week or proceed to the next step.
  c. Post GEM Incubation Cleanup.
    ***Note:*** This step is important for removing leftover biochemical reagents and unused barcodes from the post GEM reaction mixture by using Dynabeads and Solid Phase Reversible Immobilization (SPRI) beads, respectively.
    Protocol:
    i. Add Recovery Agent to each sample (DO NOT pipette mix).
    ii. Carefully remove and discard the Recovery Agent/Partitioning Oil (pink) from the bottom of the tube.
    iii. Prepare Dynabeads Cleanup Mix, add to the sample and incubate for a specific period as per the instruction.
    iv. Using a magnetic separator, remove the supernatant and perform two ethanol (freshly prepared 80% ethanol) washes.
    v. Immediately add Elution Solution and incubate for the time listed in the kit manual.
    vi. Using a magnetic separator, separate the supernatant and transfer it to another tube.
    vii. Add SPRIselect reagent to each sample and incubate.
    viii. Using the magnetic separator, remove the supernatant and perform two ethanol (freshly prepared 80% ethanol) washes.
    ix. After removing from the magnet, immediately add Elution Solution and incubate for the time listed in the kit manual.
    x. Using a magnetic separator, separate the supernatant and transfer it to another tube.
      **Pause point:** Store at 4°C for up to 72 h or at −20°C for up to 2 weeks or proceed to the next step.
  d. Library Construction:
    ***Note:*** The objective of this step is to construct the final libraries containing the P5 and P7 sequences (used in Illumina bridge amplification).
    i. Prepare Sample Index PCR Mix (as per the user guide) and add it to the sample.
    ii. Add individual Single Index primers to each well and record assignment.
    iii. Incubate in a thermal cycler (please refer to the user guide for the incubation temperatures and time required).
    iv. After incubation add the SPRIselect reagent to each sample and incubate again for 5 min.
    v. Using a magnetic separator, transfer the supernatant to a new strip tube.
    vi. Again, add SPRIselect reagent to each sample and incubate again for 5 min.
    vii. Using the magnetic separator, remove the supernatant and perform two ethanol (freshly prepared 80% ethanol) washes.
    viii. After removing from the magnet, immediately add Buffer EB, pipette mix and incubate for the time listed in the kit manual.
    ix. Using a magnetic separator, transfer the supernatant to a new strip tube.
      (The sample is now ready for post library construction quality control and sequencing).
    x. Measure the DNA concentration (ng/μL) of the sample with *Qubit* and determine the average fragment size.
      ***Note:*** We use Agilent *TapeStation* High Sensitivity D1000 ScreenTape or Agilent Bioanalyzer High Sensitivity DNA chip to determine the average fragment size.
    xi. Calculate the dsDNA library concentration (from ng/μL to nM) using the following formula:
      $$Concentration\;\left(nM\right)=\frac{\text{concentration }\left(\frac{\text{ng}}{\text{μL}}\right)}{660\left(\frac{\text{g}}{\text{mol}}\right)\text{∗ average }\;\text{library }\;\text{size }\;\left(\text{bp}\right)}*\;1000000$$
    xii. Normalize the samples to an appropriate concentration for library pooling before sequencing.
      ***Note:*** Library loading concentration differs with the instrument, consult sequencing instrument manual for additional details.
      A representative information sheet is shown below for better understanding.

**Table:** 

Sample name (lab assignment)	snATAC DNA Conc (ng/μL)	Well ID (index primer)	Average size of the library (bp)	dsDNA library Conc (nM)	DNA for 50 nM Conc (total volume 20 μL)	Water (Nuc free) for 50 nM conc (total volume 20 μL)	1:10 dilution	Further dilution
22	42.4	B5 (Single index Plate Kit N Set A)	321	200.13	5.0	15.0	5 μL DNA in 45 μL water	See the optimum loading conc of the instrument
    xiii. Pool libraries and dilute as necessary (to the appropriate concentration in nM/pM, e.g., final loading concentration for *NovaSeq*^*TM*^ is 300 nM).
      ***Note:*** Check the instrument manual for the final pool volume and concentration.
      **Pause point:** Store at 4°C for up to 72 h or at −20°C for long-term storage.

5. Sequencing:

**Table:** 

Sample name	Kit used	Well ID	Sequencing platform	Types of runs	Read length	No. of reads (M) (nuclei No.∗25,000 read pairs)	Index name	Organism	Remarks
22	Chromium Next GEM Single Cell ATAC Reagent Kits v1.1	B5	Novaseq 6000 (version S4)	Paired-end, dual indexing	150	300	i7	Human	None

### Part two: Data analysis section

**Timing: ∼2–3 h per samplefor generating chromatin accessibility counts matrix using cellranger-atac. Runtime varies based on the size of the input files and the available computational resources. We suggest running each sample in parallel if a high-performance computing cluster is accessible**

**Timing: ∼20–30 min per samplesfor creating ArrowFiles. Using multithreading will significantly reduce the processing time**

**Timing:∼20–30 minfor snATAC-seq data preparation and object conversion**

**Timing: ∼2.5 hfor pseudo-bulk replicates and calling peaks**

**Timing: less than 20 min, depending on the sample sizesfor conversion of the ArchRProject to SeuratObject and further processing**

**Timing: 3–5 min per sampleto add a gene score/activity matrix to SeuratObject**

**Timing: ∼100 minfor Signac cell-type identification with reference mapping**

**Timing: ∼ 4–5 working daysfor advanced secondary analysis**

The objective of part two is to align the sequencing data for snATAC-seq to the reference genome, obtain chromatin accessibility count matrix and process it for downstream analysis including identification of differential accessibility regions and other advanced secondary analyses.

6. Generating chromatin accessibility counts matrix using cellranger-atac.
  ***Note:*** There are two main approaches to process sequencing data produced from Chromium snATAC libraries: 10× Genomics Cloud Analysis or cellranger-atac software. Please refer to 10× Genomics’ instructions for the Cloud analysis.
  ***Note:*** Here we demonstrate how to use the cellranger-atac count pipeline to filter reads, align to a reference genome, count cell barcodes, identify transposase cut sites, detect chromatin accessibility peaks, generate chromatin accessibility counts matrix, and perform preliminary clustering analysis.
  ***Note:*** Before proceeding, please refer to the Fastq input data format.
  ***Note:*** Please see the following example of sample format from our dataset:
  Sample-100_S5_L008_I1_001.fastq.gz
  Sample-100_S5_L008_R1_001.fastq.gz
  Sample-100_S5_L008_R2_001.fastq.gz
  Sample-100_S5_L008_R3_001.fastq.gz
  Sample-43_S1_L008_I1_001.fastq.gz
  Sample-43_S1_L008_R1_001.fastq.gz
  Sample-43_S1_L008_R2_001.fastq.gz
  Sample-43_S1_L008_R3_001.fastq.gz
  a. Input and output files’ path for cellranger-atac:
    >Input_path='Your_Fastq_Input_path/'
    >Output_path='Your_Project_Output_path/cellranger_atac_output/'
  b. Path to reference annotation:
    >annotation='Your_atac_annotation_Path/refdata-cellranger-arc-GRCh38-2020-A-2.0.0/'
  c. Use cellranger-atac count to generate single-nucleus accessibility counts:
    >cellranger-atac count \
     --id=$Output_path/Your_Sample \
     --reference=$annotation \
     --fastqs=$Input_path \
     --sample=$Your_Sample_Name \
     --localcores=32 \
     --localmem=128

7. Creating ArrowFiles and an ArchRProject from cellranger-atac output and constructing TileMatrix using ArchR pipeline.
  We create ArrowFiles for each sample to reduce memory usage in R. An ArrowFile is simply a path to access information related to the individual sample, and ArchR will update the ArrowFile with additional information.
  ***Note:*** Here is a good time to refer to the ArchR website.
  ArchR accepts fragments files from cellranger-atac’s output to create ArrowFiles and an ArchRProject.
  a. **Construct the input file path of fragments samples.** For the >createArrowFiles() function in the next step, we will provide the sample fragment files, saved in the format of ‘fragments.tsv.gz’, for the required arguments, inputFiles and sampleNames. In our tutorial, sequencing outputs have a pattern as “Sample-” followed by a number, such as Sample-100, Sample-101, and Sample-10.
    >cellranger_dir='Path_to_cellranger/cellranger_atac_output'
    >sample_names=dir(cellranger_dir)[grepl('Sample',dir(cellranger_dir))]
    >sample_files=paste0(cellranger_dir, sample_names, '/outs/fragments.tsv.gz')
  b. **Create ArrowFiles.** ArchR by default generates a ‘TileMatrix’ of genome-wide 501-bp bins that makes quantifying accessible peaks easier as their length is fixed. We use the default 501-bp size, since ArchR suggests that the majority of peaks in snATAC-seq are less than 500-bp (Granja et al., 2021). Based on our previous testing, we have found it provides enough resolution for cell-type identification. However, the size of the tile matrix can be adjusted in one of the two options: 1) Reconstruct the tile matrix by adjusting the tile size when creating ArrowFiles. For example, if we want to create a TileMatrix with only 200-bp, we can adjust >creatArrowFiles(TileMatParams = list(tileSize = 200)). 2) Add the new TileMatrix to ArchRProject by computing counts for new fixed-width tile using >addTileMatrix(input = ArchRProject, tileSize = 200). ArchR also provides a ‘GeneScoreMatrix’ to store predicted gene expression based on weighting insertion counts in tiles near a gene promoter.
    >library(ArchR)
    >addArchRGenome("hg38") # Setting default genome to Genome Reference Consortium Human Build 38 Organism: Homo sapiens (human)
    # >addArchRThreads(1) # This could be a fix if issue happens due to multi-threading
    >ArrowFiles <- createArrowFiles(
     inputFiles = sample_files,
     sampleNames = sample_names,
     minTSS = 1, # Do not set this too high because you can always increase later
     minFrags = 500,
     addTileMat = TRUE,
     addGeneScoreMat = TRUE
    )
  c. **Per-cell quality control.** A stringent quality control (QC) is vital to minimize the contribution of low-quality data. In the previous step, we set lenient cutoffs for the minimum number of mapped ATAC-seq fragments required per cell (minFrags) and the minimum numeric transcription start site enrichment score (minTSS) so that we can initially assess the quality of the data. For each sample, ArchR automatically generates a cell density heatmap of TSS enrichment score and number of ATAC-seq fragments, and we provide examples in Figures 4A–4C to help demonstrate how to determine the filtering cutoff in step 7h after creating the ArchRProject using > ArchRProject().
  d. **Inferring snATAC-seq doublets with ArchR.** During the cell barcoding process, the nanodroplet can encapsulate more than one cell, and due to this technical mistake, they are considered as a single nucleus.
    >doubScores <- addDoubletScores(
     input = ArrowFiles,
     k = 10, # Refers to how many cells near a "pseudo-doublet" to count.
     knnMethod = "UMAP", # Refers to the embedding to use for nearest neighbor search with doublet projection.
     LSIMethod = 1
    )
  e. **Create an ArchRProject for your study.** ArchRProject is the data structure to hold the data and to perform all the downstream analysis.
    >proj1 <- ArchRProject(
     ArrowFiles = ArrowFiles,
     outputDirectory = "/Path_to_YourProject/ArchR/", #Output directory for ArchRProject and ArrowFiles
     copyArrows = TRUE #This is recommended so that if you modify the Arrow files you have an original copy for later usage.
    )
    **Pause point:** Saving and loading ArchRProject.
    ***Note:*** If you want to pause at any time, please save your R session in ‘.rda’ or save your ArchRProject using the following command.
    >saveArchRProject(ArchRProj = proj1, outputDirectory = "/Path_to_YourProject/ArchR/", load = FALSE)
    ***Note:*** ArchRProject can be reloaded by using the following command:
    >proj1 <- loadArchRProject(path = "/Path_to_YourProject/ArchR/", force = FALSE, showLogo = TRUE)
  f. **Add metadata to ArchRProject.** We can supply sample information (metadata), such as batch number, gender, etc, to annotate the cells. Sample IDs in the metadata file should match those in the ArchRProject.
    # Read in sample metadata:
    metadata <- read.csv('/Path/metadata.csv')
    # Add metadata to cellColData of ArchRProject
    # In our example, ArchRProject Sample information in cellColData matches the SampleName in metadata
    >for(meta in names(metadata)){
     proj1@cellColData[[meta]] <-metadata[match(as.character(proj1@cellColData$Sample), metadata$SampleName), meta]
    }
  g. **Filter doublets from ArchRProject.** Please refer to the function >filterDoublet() and the default argument filterRatio = 1 for more information if stringent filtering is needed.
    >proj1 <- filterDoublets(proj1)
  h. **Additional quality control removal of cell outliers.** TSS enrichment score is calculated based on fragment ratios centered at TSS to fragments in TSS flanking regions. In Figure 4, many cells in Sample 1 (panel A) have a relatively lower TSS enrichment score and a lower number of fragments compared to Samples 2 and 3 (panels B-C). If a significant number of nuclei from a sample have a low TSS enrichment score, it indicates poor quality of the snATAC-seq experiment. Additionally, the total number of peak fragments (nFrag) reflects the sequencing depth and cellular complexity. Nuclei with very few reads can be excluded due to low sequencing depth. Meanwhile, nuclei with an extremely high number of fragments may represent doublets, if not filtered by >filterDoublet(), or other artifacts.
    >proj1 <- proj1[which(proj1$TSSEnrichment > 2 & proj1$nFrags > 3000 & proj1$nFrags < 30000)]

8. snATAC-seq data preparation and object conversion.
  Due to the sparsity of the snATAC-seq data, standard dimensionality reduction methods cannot be used. Instead, a layered dimensionality reduction approach called latent semantic indexing (LSI) is used with snATAC-seq data. LSI uses combined steps of term frequency-inverse document frequency (TF-IDF), a normalization method that can be used to process sparse data, and singular value decomposition (SVD), a dimensionality reduction method, to assess how important a peak is to a sample.
  a. Dimensionality reduction with snATAC-seq.
    >proj1 <- addIterativeLSI(
     ArchRProj = proj1,
     useMatrix = "TileMatrix",
     name = "IterativeLSI",
     iterations = 2,
     clusterParams = list(#See Seurat::FindClusters
     resolution = c(0.2),
     sampleCells = 10000,
     n.start = 10
     ),
     varFeatures = 25000, # default
     dimsToUse = 1:30
    )
    ***Optional:*** If strong batch effect differences are observed when plotting with >plotEmbedding() from ArchR, batch effect correction with Harmony can be implemented by using >addHarmony() in ArchR. The name of the column in cellColData, a matrix containing the data associated with each nucleus, can be used to group cells together for Harmony batch correction, such as ‘Batch’ or ‘Sample’.
    >proj1 <- addHarmony(
     ArchRProj = proj1,
     reducedDims = "IterativeLSI",
     name = "Harmony",
     groupBy = "Batch")
  b. **Single nucleus clustering.** Clustering is standard practice for both snRNA-seq and snATAC-seq for cell-type grouping and to illustrate transcriptomic and epigenomic signatures of each cell type. Most single-nucleus clustering methods compute the nearest neighbor to identify communities of cell clusters. We can cluster cells in ArchR using >addClusters().
    ***Note:*** Please be aware that a change is needed for the argument, reducedDims, in both functions (addHarmony and addClusters) if a different dimensionality reduction was applied, e.g., ‘IterativeLSI’ or ‘Harmony’.
    # Clustering using Seurat’s >FindClusters() function
    >proj1 <- addClusters(
     input = proj1,
     reducedDims = "IterativeLSI",
     method = "Seurat",
     name = "Clusters",
     resolution = 0.8 #Default
    )
  c. **Single-nucleus cluster embedding.** Embeddings such as Uniform Manifold Approximation and Projection (UMAP) or t-distributed stochastic neighbor embedding (t-SNE) are used to visualize nuclei in a low-dimensional space. The primary difference between UMAP and t-SNE is the interpretation of distance between the cluster communities. Both UMAP and t-SNE maintain the local cluster community structure. However, UMAP also preserves most of the global structure in data, which means clusters near each other in UMAP are informative, compared to their location in t-SNE.
    #Uniform Manifold Approximation and Projection (UMAP)
    >proj1 <- addUMAP(
     ArchRProj = proj1,
     reducedDims = "IterativeLSI",
     name = "UMAP",
     nNeighbors = 30, #Default
     minDist = 0.1,
     metric = "cosine"
    )
    >addUMAP()computes the UMAP embedding and adds it to ArchRProject. Code for visualization is shown later in step 9, where we present two options for cell-type identification.
  d. **Pseudo-bulk replicates and calling peaks.** ArchR combines a group of similar nuclei to form a pseudo-bulk sample. The pseudo-bulk replicates overcome the sparsity problem of the snATAC-seq data for peak calling, which identifies the area in a genome that has been enriched. With the pseudo-bulk replicates generated, we can call peaks to identify accessible regions in chromatin. A peak set containing annotation for each peak is created and saved to ArchRProject.
    i. Create pseudo-bulk replicates by grouping nuclei by cluster to overcome the binary (open or close) phenomenon of chromatin accessibility.
      >proj1 <- addGroupCoverages(ArchRProj = proj1, groupBy = "Clusters")
      # Clusters was defined by the labels generated from addClusters() function
    ii. Call peaks with MACS version 2. Model-based Analysis of ChIP-Seq (MACS) version 2 currently is the default peak caller of the ENCODE ATAC-seq pipeline. ArchR pipeline uses MACS2 with the function >addReproduciblePeakSet() to produce peaks with a 501-bp fixed-width.
      >pathToMacs2 <- findMacs2()
      >proj1 <- addReproduciblePeakSet(
       ArchRProj = proj1,
       groupBy = "Clusters",
       pathToMacs2 = pathToMacs2
      )
      # add peaks matrix
      >proj1 <- addPeakMatrix(proj1)
      ***Optional:*** In addition to assigning cell identity through gene scores, ArchR allows us to align cells from a snATAC-seq dataset with cells from a snRNA-seq dataset by using >FindTransferAnchors() function from the Seurat package.
  e. **Object conversion from ArchR to Signac.** Both ArchR and Signac are popular snATAC-seq analysis packages with a comparable set of features. Some software functions are constantly under development and likely to change over time. Users can use only the Signac or ArchR pipeline during the whole analysis process. However, in this protocol, we provide an option to help with data formatting from ArchRProject to Signac SeuratObject, retaining the fixed-width peak matrix for its advantage in computation, as peak length does not need to be normalized in the downstream analysis. After data conversion, researchers can access additional analysis pipelines and other customized functions for the analysis, such as label transfer, co-accessibility analysis, and trajectory analysis.
    i. Load our object conversion package ArchRtoSignac.
      >library(ArchRtoSignac)
    ii. Set cellranger-atac directory (with fragments files).
      >fragments_dir <-'/Your_path_to_cellranger_atac_output/'
    iii. Get the gene annotation, which includes information related to genomic locations and their associated annotations, from Ensembl database in GRanges Object for the SeuratObject, using ArchRtoSignac GitHub wrapper function >getAnnotation(). Since the function utilizes an in-house function >GetGRangesFromEnsDb() from the Signac package to get gene annotation, the reference provided must be an Ensemble reference genome. >getAnnotation()also changes the annotation to UCSC genome style, which is used by both ArchR and Signac.
      ***Note:*** These arguments will be used when running this function:
      Reference: An Ensembl genome reference used for the function GetGRangesFromEnsDb to extract gene annotations from EnsDb (for example, EnsDb.Hsapiens.v86).
      seqStyle: Changes the sequence style of the annotation extracted from EnsDb to ‘UCSC’ since Signac maps to hg38.
      refversion: The assembly release and versions of the UCSC genome reference (for example, ‘hg38’).
      >annotations <- getAnnotation(
       reference = EnsDb.Hsapiens.v86,
       seqStyle = 'UCSC', refversion = 'hg38')
    iv. Obtain the fixed-width peak matrix from ArchRProject and changing the row names of the peak matrix to their matched chromosome range using ArchRtoSignac GitHub wrapper function >getPeakMatrix().
      >pm <- getPeakMatrix(ArchRProject= proj1)
    v. Make a list of the samples.
      >samples <- unique(proj1@cellColData$Sample)
    vi. Convert the ArchRProject to SeuratObject by creating a list of Seurat objects for each sample with their corresponding peak matrix and then merging objects from each sample with the ArchRtoSignac wrapper function >ArchR2Signac()
      ***Note:*** These arguments will be used when running this function:
      ArchRProject: Input ArchRProject.
      samples: A unique sample list for all processed samples from ArchRProject.
      fragments_dir: A path to the cellranger-ATAC output. The directory contains all samples’ folders before '/outs/fragments.tsv.gz'.
      pm: peak matrix recently acquired from ArchRProject by using getPeakMatrix() function in the previous step.
      reference: The same Ensembl genome reference used previously for getting the annotation.
      seqStyle: The same sequence style used for getting the annotation.
      refversion: The same assembly release and versions of UCSC genome reference used for getting the annotation.
      >seurat_atac <- ArchR2Signac(
       ArchRProject = proj1,
       samples = samples, # Provide a list of unique sample
       fragments_dir = fragments_dir, # directory of the cellranger output, the folder that contains all samples
       pm = pm, # getting peak martix
       output_dir = '/outs/',
       seqStyle = 'UCSC',
       refversion = 'hg38', # write the reference version
       reference = EnsDb.Hsapiens.v86, # choose the EnsDb as the reference
       annotation = annotations
      )
    vii. Check if the cells are in the right order before adding other information to SeuratObject.
      ***Note:*** This is extremely important for downstream analysis.
      >all.equal(colnames(seurat_atac),
       gsub('#', '_', rownames(proj1@cellColData)))
      #NOTE: Ideally, this should return TRUE
    viii. Add dimension reduction, 'Harmony' and/or 'IterativeLSI', from ArchRProject to the SeuratObject using ArchRtoSignac wrapper function >addDimRed().
      Additionally, UMAP embeddings will be automatically added to Signac SeuratObject when running this step.
      ***Note:***reducedDims: The argument for the reduction dimension can be transferred from ArchRProject to Signac SeuratObject
      >seurat_atac <- addDimRed(
       ArchRProject = proj1,
       SeuratObject = seurat_atac,
       reducedDims = 'IterativeLSI' #default
      )
      #add both 'Harmony' and ‘IterativeLSI’:
      #reducedDims = c('IterativeLSI', 'Harmony')
    ix. Add gene score or gene activity matrix to SeuratObject. Gene activity matrix from Signac and gene score matrix from ArchR similarly construct expression predictions for genes by assessing their chromatin accessibility. Under the default setting, Signac calculates gene activity by summing the fragments intersecting with gene body and promoter regions. The gene score matrix in ArchR implements a comparable approach as in Signac. However, it also considers the activity of possible distal regulatory regions using an exponential weighting model, where peaks further away from the gene have lower priority. Different models’ performances will vary differently for datasets, so there is not a universal best fit approach to predict gene activity (Granja et al., 2021). There are two different options to add a gene score/activity matrix: 1) Transfer the ArchR gene score matrix to the SeuratObject. 2) Add a new gene activity matrix using Signac functions to the SeuratObject.
      # Option 1: Transfer gene score matrix from ArchR to Signac using getGeneScoreMatrix() function in the ArchRtoSignac package:
      >gsm <- getGeneScoreMatrix(
       ArchRProject = proj1,
       SeuratObject = seurat_atac)
      >seurat_atac[['RNA']] <- CreateAssayObject(counts = gsm)
      # Option 2: Add gene activity matrix using Signac:
      >gene.activities <- GeneActivity(seurat_atac)
      >seurat_atac[['RNA']]<-CreateAssayObject(counts=gene.activities)
      >seurat_atac <- NormalizeData(
       seurat_atac,
       assay='RNA',
       normalization.method='LogNormalize',
       scale.factor = median(seurat_atac$nCount_RNA)
      )
      # change the cell identity classes
      Idents(seurat_atac) <- seurat_atac$Clusters
      **Pause point:** Saving and loading SeuratProject.
      ***Note:*** Here is a good time to pause and save your SeuratObject before moving forward. The output directory can be set and created as follows.
      >data_out <- "/Output_directory/"
      >dir.create(data_out)
      ***Note:***>saveRDS() saves your SeuratObject in .rds format with Sys.Date() in the file name, and the SeuratObject can be loaded through >readRDS().
      >saveRDS(seurat_atac, paste0(data_out, 'Seuratobj_AD_atac_inprogress_',Sys.Date(),'.rds'))
      # For example: when Sys.Date() is 2022-02-14
      >seurat_atac <- readRDS(paste0(data_in, 'Seuratobj_AD_atac_inprogress_2022-02-14.rds'))

9. Signac cell-type identification.
  Option 1: Signac manual cell-type identification with chromatin accessibility.
  Using the chromatin accessibility of each cell-type at canonical cell-type marker genes is a standard practice to decipher the cell clusters’ identities. This involves manual inspection of coverage plots showing the accessibility of given genomic regions (e.g., cell-type marker genes).
  >DimPlot(
   seurat_atac,
   reduction = "umap",
   group.by = "Clusters",
   label = FALSE,
   repel = TRUE,
   raster=FALSE) + ggtitle("snATAC-seq Clusters")
  >CoveragePlot(
   object = seurat_atac,
   region = 'given_genomic_region', #CSF1R; Input Format: chr5-150086500-150087000
   extend.upstream = 10000,
   extend.downstream = 10000,
   group.by = “Clusters”
  )
  Option 2: Signac cell-type identification with reference mapping.
  ***Note:*** The second profiling option utilizes other datasets as a reference and maps snATAC-seq data onto it. We believe this method provides some advantages. Firstly, due to the sparsity of the snATAC-seq data, using a reference from a well-annotated high-quality dataset, a published resource, or an atlas would help us better interpret our data. Ideally, the reference dataset should be sequenced from the same or similar biological sample for accurate prediction. Additionally, we can integrate snATAC-seq with snRNA-seq and analyze them together, especially if unique information related subclusters are present.
  >MapQuery() transfers anchor information from a reference dataset, integrates the two datasets, and projects the query data into the coordinates of a provided reference UMAP.
  a. Find a set of anchors between the reference and query object by using >FindTransferAnchors(). Please refer to the function source page regarding the suggested selection for dimensional reduction arguments, reduction and reference.reduction.
    ***Note:*** Switching the default assay of the query dataset is needed when the query and reference datasets' default assays do not share features. We use a snRNA-seq reference dataset in our example, so we need to use gene activity score (RNA assay) in our query snATAC-seq dataset for reference mapping.
    ># Switch default assay to RNA assay
    >DefaultAssay(seurat_atac) <- 'RNA'
    >transfer.anchors <- FindTransferAnchors(
     reference = seurat_reference,
     query = seurat_atac,
     reduction='cca', #Dimensional reduction selection.
     dims = 1:30
    )
  b. Rerun the mapping process by >RunUMAP() on the selected reference dataset with the argument return.model = TRUE to compute UMAP and store the UMAP model to run >MapQuery() later. Our reference, a snRNA-seq dataset, is integrated and analyzed using Liger, which relies on integrative non-negative matrix factorization (inmf). We specified the reference reduction based on this previous integration process, so please adjust the reduction argument accordingly.
    >seurat_reference <- RunUMAP(seurat_reference,
     reduction = 'inmf',
     # reduction ='reduction_used_for_the_reference'; for example, pca, inmf and etc.
     dims = 1:30, n.neighbors=30L, min.dist=0.10, return.model=TRUE
    )
  c. For the transferring reduction, please refer to >FindTransferAnchors() function source page. We chose 'cca', a suggested reduction for the projection from snRNA-seq to snATAC-seq. Additionally, ‘lsiproject’ is advised for the projection from snATAC-seq to snATAC-seq, and ‘pcaproject’ is for the projection from snRNA-seq to snRNA-seq.
  d. Map the snATAC-seq dataset onto the provided reference using >MapQuery(), enabling cell-type label transferring from reference dataset to the query dataset.
    >seurat_atac <- MapQuery(
     anchorset = transfer.anchors,
     reference = seurat_reference,
     query = seurat_atac,
     refdata = list(cluster = "Cell.Type"),
     reference.reduction = 'inmf',
     reduction.model = "umap"
    )
    Figure 5 shows UMAPs of mapped query nuclei from snATAC-seq to a reference dataset as an example. All extracted nuclei that passed QC are shown in Figure 5A. However, due to the sparsity of the snATAC-seq data, you should choose to filter predicted cells with a desired cutoff to limit the number of false positives after using >MapQuery() for reference mapping.
    After adjusting the predicted.cluster.score parameter as shown in Figures 5B–5D, nuclei in ATAC-seq with high predicted cluster scores can be preserved for future analysis. Additionally, please be careful to not remove too many nuclei.
  e. Remove nuclei with low predicted cluster scores.
    >seurat_atac_90 <- subset(seurat_atac, predicted.cluster.score >= 0.90)
    ***Note:*** Results (Figures 6 and 7) of step 9 Signac cell-type identification are in the expected outcomes section.

10. Differentially accessible regions.
  ***Note:*** One of the standard practices in a snATAC-seq study is finding differentially accessible regions (DAR) between comparison groups by cell type. We can use the same function as in Seurat >FindMarkers() to perform a differential accessibility test. Due to the sparsity of snATAC-seq data, we would suggest lowering the minimum fraction of cells, min.pct. Furthermore, test.use can be changed as desired; however, Signac suggests using a logistic regression framework (Stuart et al., 2021), which can account for latent variables, such as technical and/or biological variables.
  a. Find the differentially accessible peaks.
    # Change the Default assay to peaks
    >DefaultAssay(seurat_atac) <- 'peaks'
    # Change the identity classes to the comparison groups for your experiment (e.g., Group1 and Group2 specified in metadata column Group)
    >Idents(seurat_atac) <- seurat_atac$Group
    >da_peaks <- FindMarkers(
     object = seurat_atac,
     ident.1 = "Group1", # For example: Disease
     ident.2 = "Group2", # For example: Control
     min.pct = 0.01,
     logfc.threshold = 0,
     test.use = 'LR',
     # test.use = 'LR': a logistic regression framework to determine differentially accessible regions
     latent.vars = 'Variables_to_test'
     # For example latent.vars = c("Batch","Age","Sex","PMI"), used only when test.use is one of 'LR', 'negbinom', 'poisson', or 'MAST'
    )

11. Advance secondary analysis.
  a. Pseudotime trajectory analysis using Monocle3.
    During development, disease progression, or in response to environmental or drug stimuli, cells may transition between different states, which likely have different genetic or epigenetic signatures, due to genes being silenced or newly activated. Monocle3, originally created for snRNA-seq data, can detect gene expression dynamics and trajectory over time within cell types. We can, however, utilize Monocle3 to perform trajectory analysis for snATAC-seq trajectory with the help of its extension Cicero, where single-nucleus chromatin accessibility changes place each cell in a predicted position in trajectory (Pliner et al., 2018; Cao et al., 2019). Afterward, differential accessibility analysis can be performed to determine changes in chromatin accessibility across the cell states. Depending on the goals of your studies, we can perform trajectory analysis on cell lineages or a cell-type (for example, microglia), and we can do this by subsetting the cell clusters. In order to use Monocle3 for trajectory analysis, the SeuratObject needs to be converted to a cellDataset object using >as.cell_data_set().
    i. >as.cell_data_set() is a SeuratWrapper function. Install SeuratWrappers and its dependencies first before using the conversion function.
      >remotes::install_github('satijalab/seurat-wrappers')
      >library(SeuratWrappers)
    ii. Convert the group of nuclei you wish to study from snATAC-seq SeuratObject to cellDataset object.
      >cd <-as.cell_data_set(seurat_atac.CellsofInterestGroup)
    iii. In order to compute pseudotime estimates for a trajectory, a start node or root needs to be identified, which we can do with >get_earliest_principal_node(). We can also select cells through the interactive function in Monocle3 using >order_cells() without specifying the root nodes or root cells. Additionally, we can use pre-selected cells if the start is known by supplying the parameter, >root_cells = "a vector of pre-selected cells", in >order_cells().
      ***Note:*** Please refer to Monocle3’s documentation for the helper function used for identification of root principal points.
    iv. Calculate the measurement of cell progression in pseudotime.
      >cd<-order_cells(cd, root_pr_nodes=get_earliest_principal_node(cd))
  b. Cis-regulatory network analysis using Cicero.
    One important piece of information from snATAC-seq data is the linkage of candidate cis-regulatory elements to their potential target genes, and this provides an opportunity to investigate common and specific regulatory mechanisms across different cell types. This linkage is known as co-accessibility, the correlation of accessibility between two peaks. Cicero computes the correlation between all pairs of peak sites within 500 kb with a distant-dependent penalty and therefore provides co-accessibility scores for pairs of peaks in snATAC-seq data (Pliner et al., 2018). Additionally, Cicero groups highly co-accessible groups of peaks into “cis co-accessibility networks” (CCANs), which are modules of highly co-accessible sites. Co-accessible peaks in CCANs may be physically close to form a chromatin hub, where they interact with a common set of transcriptional factors in a loop (Pliner et al., 2018).
    i. We use Cicero, which is an extension of Monocle3, previously used for trajectory analysis, to perform cis-regulatory network analysis, and therefore, the SeuratObject needs to be converted to a cellDataset object using >as.cell_data_set() as in trajectory analysis. However, it then needs to be further made into a Cicero object by using >make_cicero_cds().
      >cd <- as.cell_data_set(seurat_atac.CellsofInterestGroup)
      >atac.cicero <- make_cicero_cds(cd, reduced_coordinates = reducedDims(cd)$UMAP)
    ii. Select the genomic region of interest from the SeuratObject, and convert the chromosome sizes to a data frame before running Cicero to find co-accessibility.
      >conns <- run_cicero(atac.cicero, genomic_coords = genome.df, sample_num = 100)
    iii. Identify cis co-accessibility networks (CCANs).
      >ccans <- generate_ccans(conns)
      ***Note:*** Please refer to the Cicero function in Signac and Cicero for Monocle3 for more detailed information and visualization.
  c. DNA sequence motif and transcription factor footprinting analysis.
    After identification of differentially accessible peaks, we may also want to determine the binding activities of transcription factors, especially to those at accessible chromatin sites. Signac provides two separate and complementary approaches for performing motif analysis: one by finding the overrepresented motifs in the set of differentially accessible peaks we found in Part Two step 10, and another one by computing per-cell motif activity scores using chromVAR (Schep et al., 2017) and then identify differentially active motifs using >FindMarkers().
    i. Identify overrepresented motifs.
      >enriched.motifs <- FindMotifs(object = seurat_atac, features = da_peaks)
    ii. Compute and identify differential motif activities.
      #>BiocManager::install("BSgenome.Hsapiens.UCSC.hg38")
      >seurat_atac <- RunChromVAR(
       object = seurat_atac,
       genome = BSgenome.Hsapiens.UCSC.hg38
      )
      >DefaultAssay(seurat_atac) <- 'chromvar'
      >differential.activity <- FindMarkers(
       object = seurat_atac,
       ident.1 = "Group1", # For example: Disease
       ident.2 = "Group2", # For example: Control
       only.pos = TRUE,
       mean.fxn = rowMeans,
       fc.name = "avg_diff"
      )
      We can then examine the footprints of the overrepresented motifs or motifs with differential activity by using >Footprint(). Transcription factor footprinting allows us to predict the precise binding location of a TF at a particular locus.
  d. Genomic regions enrichment of annotations analysis.
    Coding regions with corresponding gene names are usually well documented with their biological functions; however, non-coding regions comparatively lack this information, especially at distal binding sites. GREAT maps cis-regulatory elements to neighboring genes within 5 kb upstream and 1 kb downstream of the TSS to predict their functions based on functional annotation databases and therefore associates cis-regulatory elements to not only their proximal binding events.
    GREAT (McLean et al., 2010) is available online and can be used in R with >library(rGREAT). Please be aware that the default reference is hg19 in rGREAT version 4, and the reference genomes can be changed through species = "hg38" or "mm10" in >submitGreatJob().
    >job = submitGreatJob(bed, species = "hg38")
    ***Note:*** Please refer to the rGREAT tutorial for more details.

**Figure 3:**
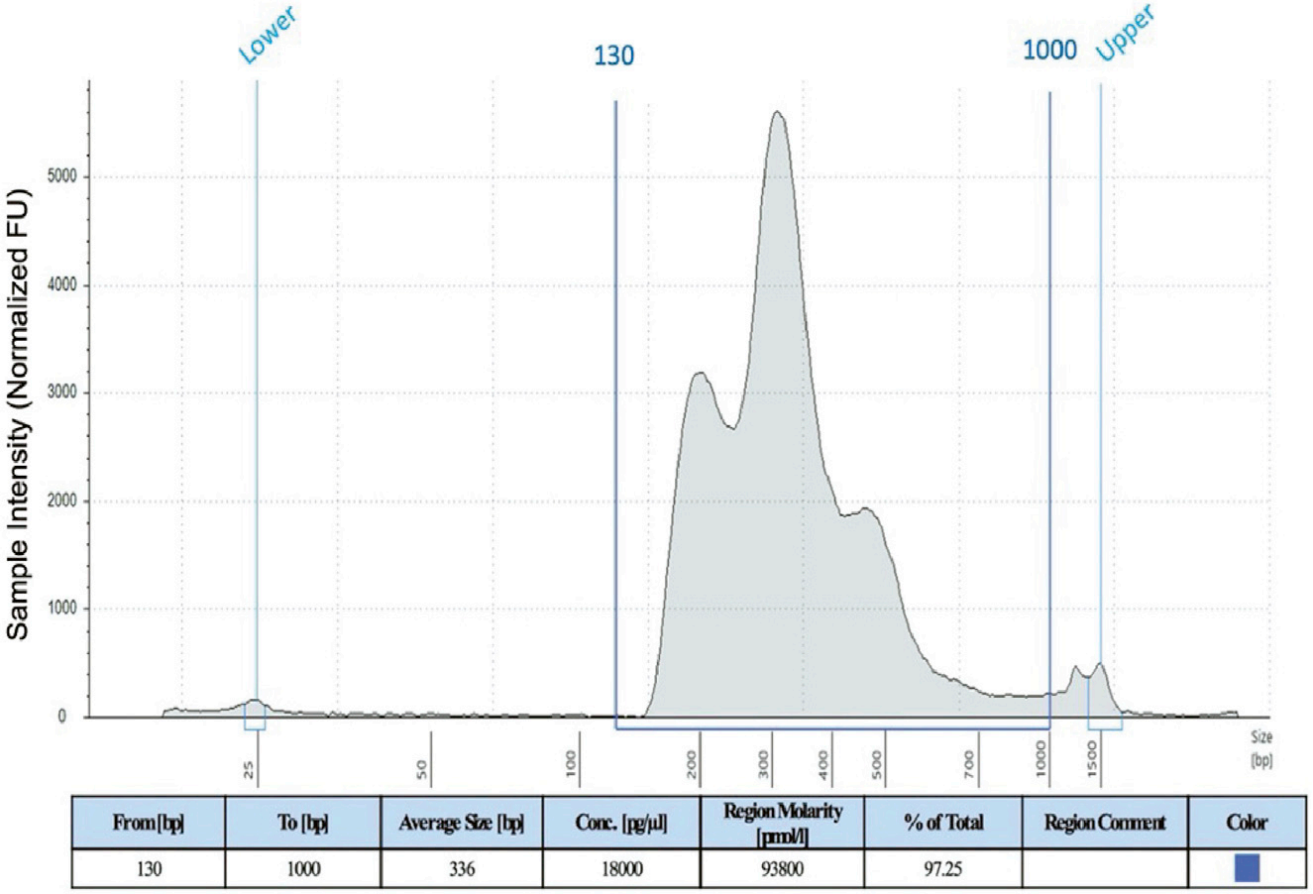
Representative trace A representative *TapeStation* profile from 50 mg of frozen human brain tissue (PFC). 2 μL of DNA from each sample (from step 4.d.ix) was mixed with 2 μL of High Sensitivity D1000 Sample Buffer followed by vortex and brief centrifugation. The ladder and the samples were then loaded into the *TapeStation* instrument to run.

**Figure 4:**
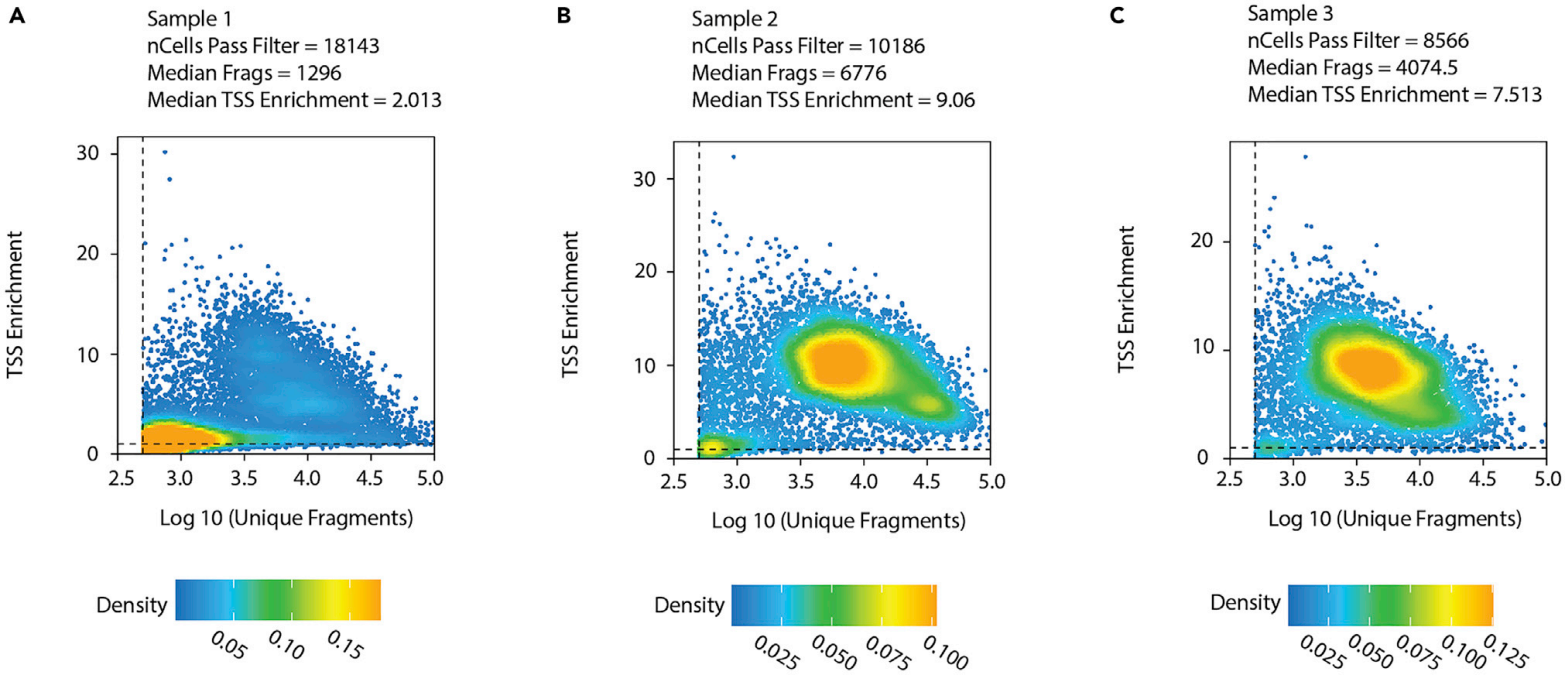
Example output of per-cell quality control Example of per-cell QC as output by ArchR. (A) In Sample 1, a high percentage of nuclei have low TSS enrichment scores and low fragments numbers. (B and C) In Samples 2 and 3, most of the nuclei have good TSS enrichment scores and fragments numbers. However, further filtering is still needed to remove outliers that are low quality (potential doublets or artifacts).

**Figure 5:**
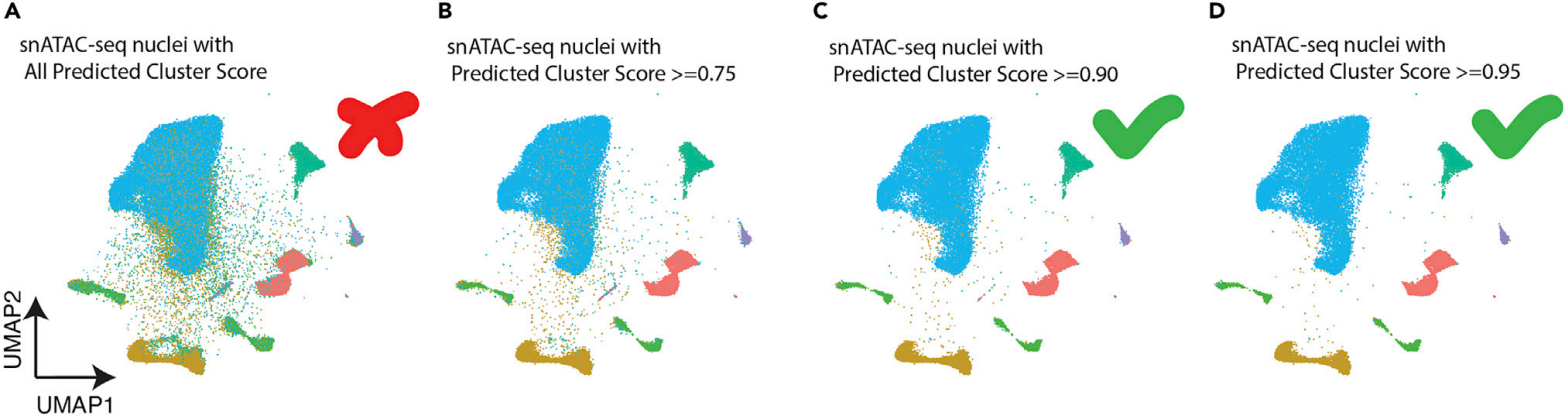
Example output of MapQuery predicted clusters with different cutoff scores Predicted cluster scores were obtained from mapping snATAC-seq onto a reference dataset. (A–D) Example UMAPs showing all nuclei (A), nuclei with predicted cluster score >= 0.75 (B), nuclei with predicted cluster score >= 0.90 (C), and nuclei with predicted cluster score >= 0.95 (D).

**Figure 6:**
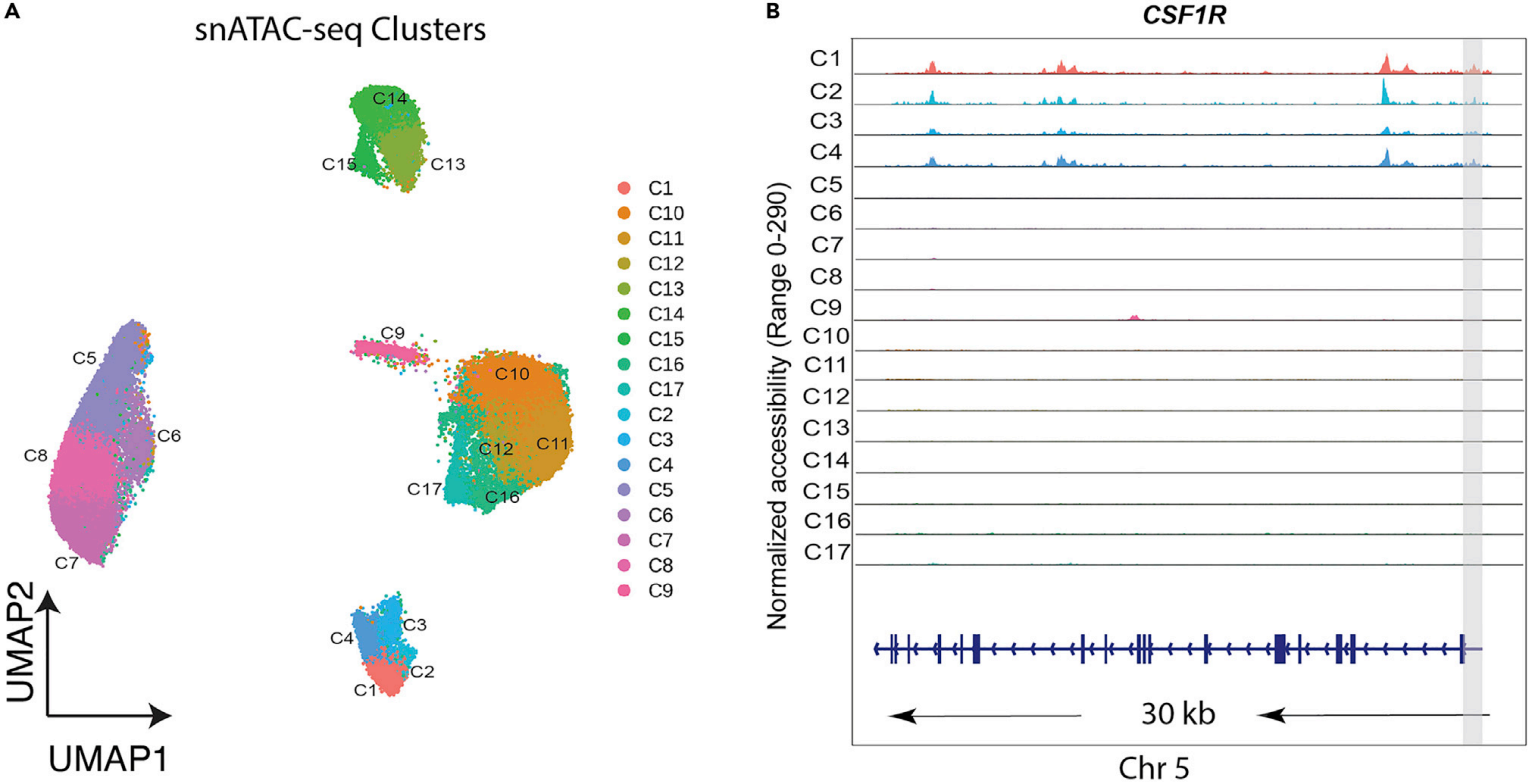
UMAPs of snATAC-seq clusters and coverage plots for cell-type identification (A) Cell clusters from snATAC-seq identified in ArchR pipeline. (B) Coverage plots of CSF1R in each cell cluster shows the pseudo-bulk chromatin accessibility profiles over a 30 kb genomic region to facilitate cell-type identification. The promoter/ TSS is highlighted in gray with gene model and chromosome position shown below. Gene with chromosome position: CSF1R (chr5: 150086500–150087000) for microglia.

**Figure 7:**
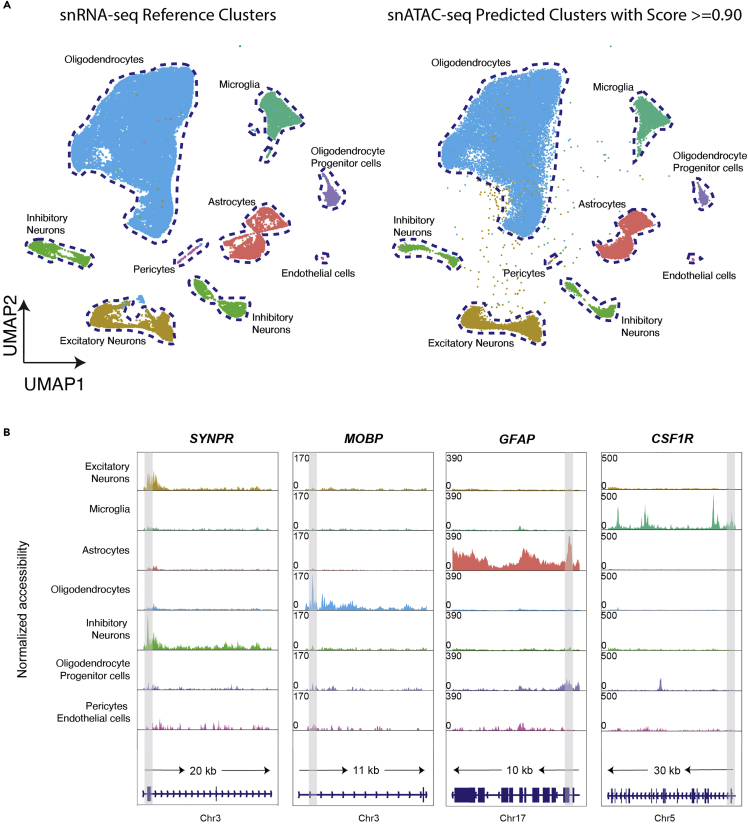
snRNA-seq identified cell-types as reference and snATAC-seq MapQuery predicted cell-types (A) Cell-type of each cluster is predicted, and cell-type labels are transferred from reference snRNA-seq data (Morabito et al., 2021). (B) Examples of pseudo-bulk chromatin accessibility profiles for neurons, oligodendrocytes, astrocytes, and microglia at their canonical cell-type marker genes. The promoter/TSS is highlighted in gray with gene model and chromosome position shown below. Gene with chromosome position: SYNPR (chr3: 63278010–63278510) for neurons, MOBP (chr3: 39467500–39468000) for oligodendrocytes, GFAP (chr17: 44915000–44915500) for astrocytes and CSF1R (chr5: 150086500–150087000) for microglia

## Expected outcomes

The tissue dissection protocol and modified single nucleus isolation protocol should give rise to enough intact nuclei from the frozen brain to achieve targeted nuclei recovery for further processing including library generation (Please refer to Figure 2B). The kit-based library generation process is highly reproducible and provides good quality libraries (Please refer to Figure 3) for sequencing.

Figure 6Ashows identified snATAC-seq clusters from the ArchR pipeline after running step 8.b that are then embedded into a UMAP by running step 8.c and plotted as demonstrated in step 9 Option 1. Clusters C1, C2, C3, and C4 have significant chromatin accessibility at the promoter/TSS and gene body of CSF1R, a canonical cell-type marker for microglia, as shown in Figure 6B. This result indicates that this group of cell clusters is microglial. Researchers should be able to clearly distinguish cell-type clusters after using known cell-type markers for identification.

Figure 7Ashows a UMAP of the reference snRNA-seq dataset and projected cell-types in snATAC-seq query dataset with a prediction score larger than 0.90. Researchers should be able to clearly distinguish cell-type clusters after reference mapping and filtering outlined in Part Two step 9 Option 2 a–e. Figure 7B shows snATAC-seq pseudo-bulk chromatin accessibility profiles of canonical cell-type marker genes (SYNPR for neurons, MOBP for oligodendrocytes, GFAP for astrocytes and CSF1R for microglia) as validation of the accuracy of MapQuery for label transfer and cell-type prediction.

## Limitations

### Part one: Wet lab

The authors do not recommend substituting buffers or buffer components/reagents by other providers. Additionally, the protocol is not optimized for low (<45 mg) amount of human brain tissue. Please start with 45–55 mg of tissue.

### Part two: Dry lab

Due to the sparsity of snATAC-seq data, calculations constructing the gene activity or gene score matrix cannot accurately predict gene expression level by only assessing chromatin accessibility. Improvements in expression prediction algorithms for snATAC-seq data will help predict gene expression levels, which will, in turn, help in cell-type identification.

The performance and accuracy of reference mapping for the cell-type identifications are dependent on the selection of the reference dataset (listed in step 9 Option 2 a–e). Having a well-annotated dataset collected from the same or similar brain regions for the reference dataset will significantly improve the prediction of cell-type for the clusters.

## Troubleshooting

### Problem 1

**Figure 8:**
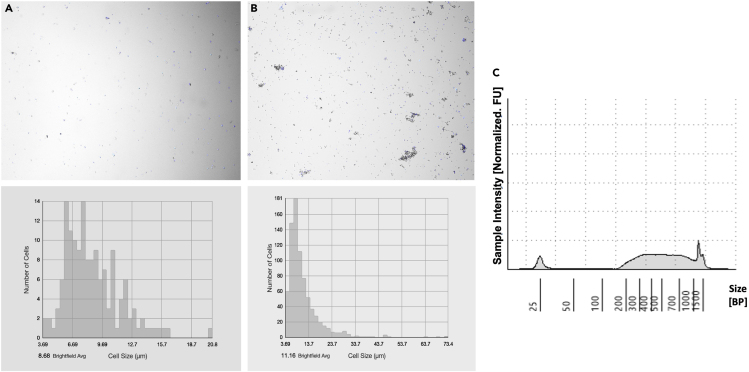
Examples of poor-quality nuclei and library profile (A and B) Representative images of samples with abnormal low no. of nuclei (95 nuclei/ul) (A) and excessive debris in isolated nuclei and their respective cell size plots (B). (C) Unusual profile in *TapeStation* (D1000).

Abnormal low No. of nuclei as shown in Figure 8A.

Step: 2.q (nuclei counting/quality check step).

### Potential solution

Start with ≥50 mg of tissue. Grind the sample to a fine powder (flour-like consistency) with a mortar and pestle in liquid nitrogen before adding the Lysis Buffer. Ensure the sample is kept frozen while grinding.

### Problem 2

Excessive debris in isolated nuclei as shown in Figure 8B.

Step: 2.q (nuclei counting/quality check step).

### Potential solution

If the resulting nuclei suspension shows debris, the filtration (through a 40 μm filter) of the nuclei suspension should be repeated.

### Problem 3

Low DNA concentration measured by *Qubit* or unusual profile in *TapeStation* High Sensitivity D1000 ScreenTape or D5000 ScreenTape (as shown in Figure 8C).

**Step: 4.d.x** (Post Library Construction QC); Sample from step **4.d.ix**.

### Potential solution

Try to start with a good-quality sample. Measure RIN or perform DNA genotyping to make sure the samples are good in quality. However, avoid pooling human samples.

Make sure all the steps were performed as per the protocol. Read the whole protocol, including the kit protocols, (with special attention to buffer and Master Mix making) before starting the experiments.

Please double check all the incubation temperatures and cycle numbers before incubation.

In addition, carefully read the troubleshooting part from the specific kit user manual.

### Problem 4

**Figure 9:**
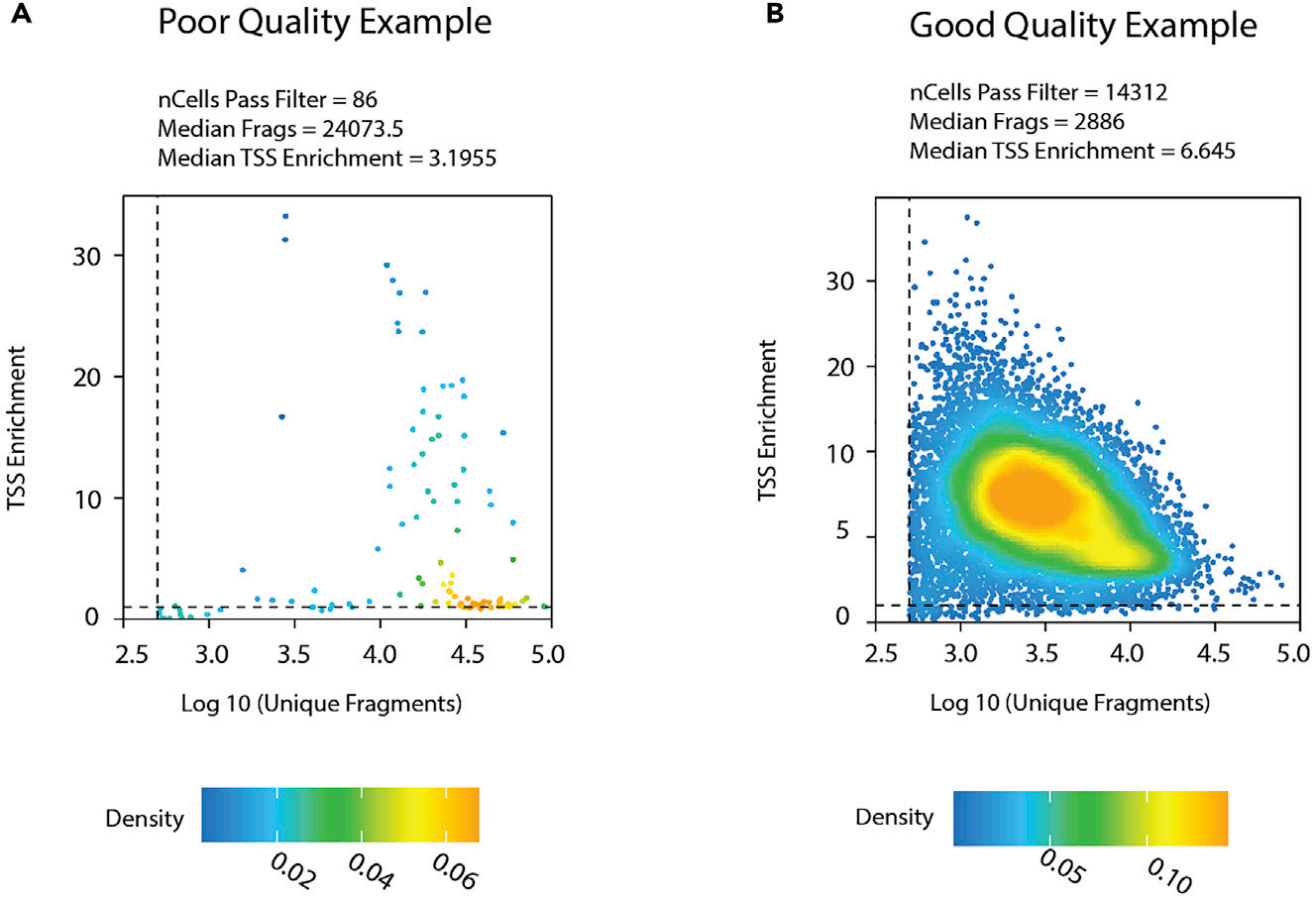
Examples of per-cell quality control from step7.c Each dot is a representation of a single nucleus. (A) An example of poor quality snATAC-seq data that has not enough nuclei to pass the per-cell quality control for statistically meaningful analysis. Extremely high median fragments count in nuclei and relatively low median TSS enrichment further confirm the poor quality of the data. (B) An example of good quality snATAC-seq data with enough nuclei and reasonable median fragments count and good median TSS enrichment for downstream analysis.

Failed QC or not enough nuclei for downstream analysis as shown in Figure 9.

Step: 7.c: Per-cell quality control.

### Potential solution

Try to start with an experimental design including multiple technical and biological replicates.

Repeat the web lab procedure on additional samples if possible.

### Problem 5

Parallel processing or multithreading error in ArchR.

Steps: 7 and 8.

Errors related to parallel processing can happen when running ArchR. However, this problem is much more related to the computer environment than ArchR. Please refer to the ArchR GitHub issue page if similar problems occur.

### Potential solution

Set the thread as 1. Using a single thread could solve most of the problems related to parallel processing. However, the disadvantage is that it may require much more time to process the same function compared to using parallel processing.

Change the thread usage of the whole session to 1.

>addArchRThreads(1)

Or change the thread usage of a function to 1. For example, in >createArrowFiles() function, you can change the default argument threads = getArchRThreads() to threads = 1.

***Note:*** Any other error related to the use of ArchR and Signac, please refer to the ArchR and Signac GitHub Issue pages.

## Resource availability

### Lead contact

Further information and requests for resources and reagents should be directed to and will be fulfilled by the lead contact, Dr. Vivek Swarup (vswarup@uci.edu).

### Materials availability

This study did not generate new unique reagents.

## Data Availability

All the multi-omics raw and processed data are available at https://www.synapse.org/#!Synapse:syn22079621/. Raw sequencing data have been deposited into the National Center for Biotechnology Information Gene Expression Omnibus: GSE174367. Please be aware the processed data deposited in Synapse were processed using an older version of cellranger for the previous AD study (Morabito et al., 2021). Examples for snATAC-seq data shown in this protocol are processed using the current version listed in the preparation section. The custom code in the R package used for this paper is available on GitHub (https://github.com/swaruplabUCI/ArchRtoSignac; DOI: 10.5281/zenodo.6612020 https://zenodo.org/record/6612020#.YppLU-zMITs), and other functions are also used for snATAC analysis are from ArchR and Signac pipeline.

## References

[bib1] Buenrostro J.D., Wu B., Chang H.Y., Greenleaf W.J. (2015). ATAC-seq: a method for assaying chromatin accessibility genome-wide. Curr. Protoc. Mol. Biol..

[bib2] Cao J., Spielmann M., Qiu X., Huang X., Ibrahim D.M., Hill A.J., Zhang F., Mundlos S., Christiansen L., Steemers F.J. (2019). The single-cell transcriptional landscape of mammalian organogenesis. Nature.

[bib3] Fang R., Preissl S., Li Y., Hou X., Lucero J., Wang X., Motamedi A., Shiau A.K., Zhou X., Xie F. (2021). Comprehensive analysis of single cell ATAC-seq data with SnapATAC. Nat. Commun..

[bib4] Granja J.M., Corces M.R., Pierce S.E., Bagdatli S.T., Choudhry H., Chang H.Y., Greenleaf W.J. (2021). ArchR is a scalable software package for integrative single-cell chromatin accessibility analysis. Nat. Genet..

[bib5] McLean C.Y., Bristor D., Hiller M., Clarke S.L., Schaar B.T., Lowe C.B., Wenger A.M., Bejerano G. (2010). GREAT improves functional interpretation of cis-regulatory regions. Nat. Biotechnol..

[bib6] Morabito S., Miyoshi E., Michael N., Shahin S., Martini A.C., Head E., Silva J., Leavy K., Perez-Rosendahl M., Swarup V. (2021). Single-nucleus chromatin accessibility and transcriptomic characterization of Alzheimer's disease. Nat. Genet..

[bib7] Ntranos V., Yi L., Melsted P., Pachter L. (2019). A discriminative learning approach to differential expression analysis for single-cell RNA-seq. Nat. Methods.

[bib8] Pliner H.A., Packer J.S., McFaline-Figueroa J.L., Cusanovich D.A., Daza R.M., Aghamirzaie D., Srivatsan S., Qiu X., Jackson D., Minkina A. (2018). Cicero predicts cis-regulatory DNA interactions from single-cell chromatin accessibility data. Mol. Cell.

[bib9] Rai V., Quang D.X., Erdos M.R., Cusanovich D.A., Daza R.M., Narisu N., Zou L.S., Didion J.P., Guan Y., Shendure J. (2020). Single-cell ATAC-Seq in human pancreatic islets and deep learning upscaling of rare cells reveals cell-specific type 2 diabetes regulatory signatures. Mol. Metabol..

[bib10] Schep A.N., Wu B., Buenrostro J.D., Greenleaf W.J. (2017). chromVAR: inferring transcription-factor-associated accessibility from single-cell epigenomic data. Nat. Methods.

[bib11] Shashikant T., Ettensohn C.A. (2019). Genome-wide analysis of chromatin accessibility using ATAC-seq. Methods Cell Biol..

[bib12] Stuart T., Srivastava A., Madad S., Lareau C.A., Satija R. (2021). Single-cell chromatin state analysis with Signac. Nat. Methods.

[bib13] Ziffra R.S., Kim C.N., Ross J.M., Wilfert A., Turner T.N., Haeussler M., Casella A.M., Przytycki P.F., Keough K.C., Shin D. (2021). Single-cell epigenomics reveals mechanisms of human cortical development. Nature.

